# Uninflamed colonic tissue harbors humoral activity linked to anti-TNF therapy non-response in ulcerative colitis

**DOI:** 10.1016/j.isci.2026.117408

**Published:** 2026-09-08

**Authors:** Gunnar Andreas Enerhaug Walaas, Silje Sveaas, Lusie Frostvoll Kuraas, Arun Sridhar, Kari Lillebråten, Hanne Fossen, Henrik Sahlin Pettersen, Arnar Flatberg, Atle van Beelen Granlund, Arne Kristian Sandvik, Ann Elisabet Østvik, Torunn Bruland, Ingunn Bakke

**Affiliations:** 1 Department of Clinical and Molecular Medicine (IKOM), NTNU - Norwegian University of Science and Technology, Trondheim, Norway; 2 Department of Gastroenterology and Hepatology, Clinic of Medicine, St. Olav’s University Hospital, Trondheim, Norway; 3 Department of Pathology, St. Olav’s University Hospital, Trondheim, Norway; 4 Clinic of Laboratory Medicine, St. Olav’s University Hospital, Trondheim, Norway

**Keywords:** precision medicine, inflammatory bowel disease, biomarker, spatial transcriptomics, WGCNA, immunoglobulins, antibodies, B- and plasma cells, laser microdissection, network analysis

## Abstract

Anti-TNF therapy is central for treating ulcerative colitis, yet ∼40% are primary non-responders. To find pre-treatment signatures distinguishing anti-TNF non-responders from responders, we did serum cytokine profiling and laser microdissection transcriptomics on paired uninflamed and inflamed colonic epithelium and lamina propria from patients matched at baseline but differing in endoscopic outcome. Although inflammation drove more epithelial changes, the signatures separating non-responders from responders were found in uninflamed lamina propria, with elevated cell-cycle genes and a co-expression network enriched for humoral immune genes consistently higher expressed in non-responders. Cellular deconvolution localized this network to IgA plasma cells, confirmed by tissue staining, and the association was validated by pseudobulk analysis of an independent single-cell atlas. Baseline epithelial signatures or serum cytokines did not differ significantly between groups. These findings point to TNF-independent humoral activity already present in uninflamed mucosa as a candidate contributor to non-response, with implications for prediction and alternative therapies.

## Highlights

• Mucosal compartment-specific signatures may predict anti-TNF non-response in UC
• Uninflamed lamina propria shows strongest baseline responder differences
• IgA plasma cells, humoral and cell-cycle programs are enriched in non-responders

## Graphical abstract

**Figure:**
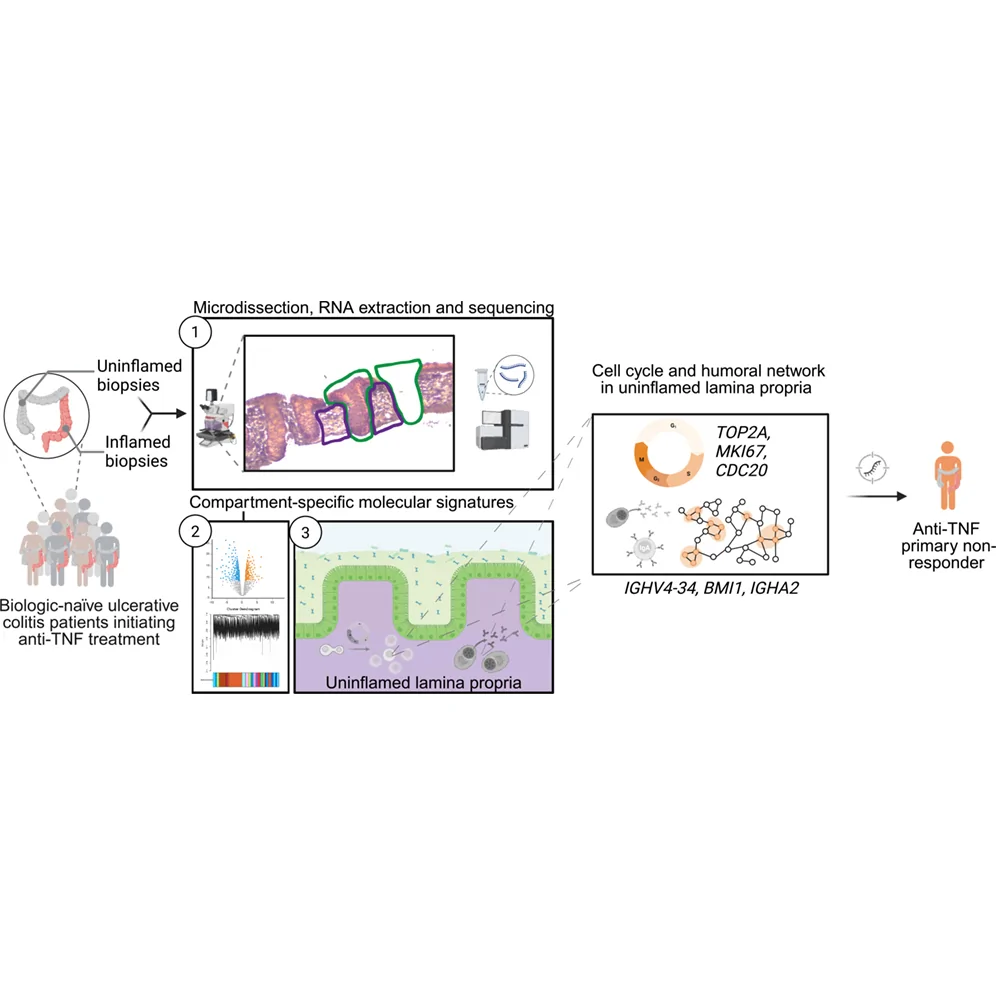

## Teaser

Health sciences; Immunology; transcriptomics

## Introduction

Ulcerative colitis (UC) is a chronic inflammatory bowel disease (IBD) that significantly impacts quality of life through persistent colonic inflammation that causes debilitating symptoms.^1^^,^^2^ Typically manifesting in early life, patients contend with the disease for decades.^2^ While colectomy remains curative, therapeutic strategies focus on inducing remission and preventing relapse.^3^ Anti-tumor necrosis factor (anti-TNF) agents are widely used for moderate-to-severe UC,^3^^,^^4^ utilizing antibodies targeting TNF, albeit through unresolved mechanisms.^5^^,^^6^ Yet, only a subset have sustained therapeutic effect, with 10–40% exhibiting primary non-response^7^ and up to 50% of initial responders developing secondary non-response.^8^ This underscores the need to enhance our understanding of the pathophysiology underlying anti-TNF response to develop precision medicine biomarkers predicting treatment outcomes.

Multiple studies have characterized histological, cellular, and molecular differences between anti-TNF responders and non-responders in UC.^9^^,^^10^^,^^11^^,^^12^^,^^13^ Arijs et al.^12^ identified 74 differentially expressed genes (DEGs), while West and Hegazy et al.^11^ demonstrated baseline oncostatin M (*OSM*) upregulation associated with non-response. Friedrich et al.^13^ identified three gene networks associated with non-response to anti-TNF, anti-integrin, and corticosteroids, comprising inflammatory fibroblasts and OSM-expressing neutrophils, or lymphoid aggregates expressing B lymphocyte chemokines (CCL19, CCL21, CXCL13). A meta-analysis revealed increased baseline plasma cells in intestinal inflamed mucosa as predictive of anti-TNF non-response in IBD.^9^ Lastly, a recent single-cell RNA sequencing study identified baseline differences between responders and non-responders in epithelial and myeloid cells.^10^

Despite promising biomarker development,^14^ clinical implementation remains limited. Most gene expression studies analyze inflamed whole mucosal samples with frequent reanalysis of identical cohorts, limiting data diversity and reliability. Studies typically compare responders and non-responders at group level with heterogeneous disease status and intestinal tissue type, including both colon and ileum and inflamed and uninflamed mucosa. Only a few have included paired uninflamed/inflamed samples.^10^^,^^13^ While single-cell analysis demonstrates advantages for identifying responder vs*.* non-responder differences,^10^ comparable studies with spatial resolution and in-depth bulk RNA sequencing remain scarce.

This study used strict criteria for patient homogeneity and included patients with UC with equal baseline disease activity, sufficient serum drug concentrations, and unambiguous response or non-response to anti-TNF therapy after 6 months. We utilized laser microdissection technology to investigate compartment-specific transcriptomics from both colonic epithelium and lamina propria in paired uninflamed and inflamed mucosal samples acquired prior to anti-TNF therapy, as well as baseline serum cytokine profiles. An independent cohort was used to validate the findings.^10^ The aim was to do a comprehensive baseline comparison of serum and colonic tissue to identify molecular signatures and potential predictive biomarkers, distinguishing future anti-TNF UC non-responders from responders before initiating treatment.

## Results

### Anti-TNF responders and non-responders have comparable baseline clinical characteristics and serum cytokine profiles

**Figure 1:**
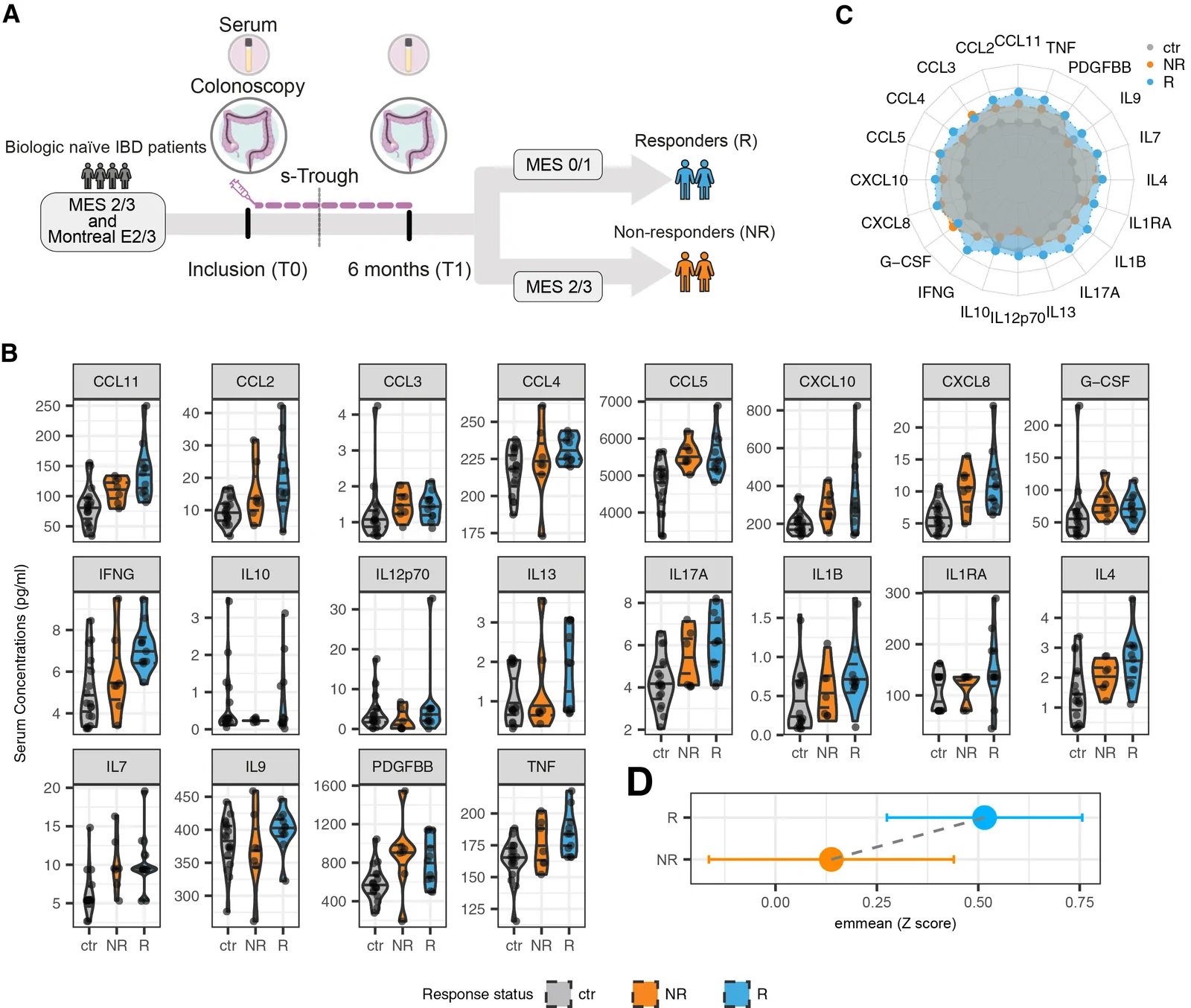
Serum cytokine profiles distinguish anti-TNF responders from non-responders (A) Colonoscopy biopsies and serum were collected from biologically naive patients with UC with active disease before anti-TNF initiation (T0) and classified as responders or non-responders based on endoscopic Mayo score (MES) changes after 6 months (T1). (B and C) Baseline serum cytokine concentrations (pg/mL) (median ± interquartile range), and (C) mean Z-score-transformed observations for non-IBD controls (ctr) (*n* = 16), NR (*n* = 8) and R (*n* = 11). (D) Linear mixed model-estimated marginal means (±95% confidence interval) of normalized *Z* score cytokine concentrations. Mean difference between R and NR: 0.378 (Padj = 0.055) performed with Student’s *t* test. Also see Figures S1 and S2.

Large inter-individual variability among patients with UC can confound disease differences between responders and non-responders to anti-TNF treatment. Therefore, we included biologically naive patients with UC (*n* = 19) with equal baseline disease activity and unambiguous endoscopic response status at 6 months (11 responders/8 non-responders) (Figures 1A, S1B, and S1C; Table 1). We found that the groups’ demographics, body mass index (BMI), serum C-reactive protein (s-CRP), medication, and the duration, extent, and severity of the disease were comparable. The minor non-responder group receiving adalimumab had lower s-trough than responders but was still above therapeutic levels. Median fecal calprotectin was lower in responders than non-responders, although this comparison was limited by more missing measurements in non-responders. A similar number of patients had calprotectin above 1000 (5 and 4, respectively).

**Table 1:** Baseline characteristics of included UC patients and non-IBD controls

	Responders (*n* = 11)	Non-responder (*n* = 8)	Non-IBD controls (*n* = 16)	*P* value
Female/Male	6/5	3/5	10/6	0.6499
Age				
Mean (SD)	35.1 (11.2)	38.5 (17.5)	53.5 (21.5)	0.6372
Min, Max	18, 52	17, 60	17, 71	–
Mean duration of disease (SD), years	6.6 (5.7)	9.2 (6.8)	N/A	0.4468
Smoking				
Yes	0 (0%)	0 (0%)	N/A	–
No	9 (81%)	5 (63%)	N/A	–
Previously	0 (0%)	1 (13%)	N/A	–
N/A	2 (18%)	2 (25%)	–	–
Mean BMI kg/m2 (SD)	24.3 (3.7)	27.9 (5.9)	N/A	0.1521
Medication type (%)			N/A	–
Infliximab	7 (64.6%)	5 (62.5%)	–	1.0
Adalimumab	4 (36.4%)	3 (37.5%)	–	1.0
Median s-trough value^b^ μg/mL (SD)			N/A	–
Infliximab	12.6 (7.7)	11.3 (0.79)	–	0.73
Adalimumab	14.4 (2.6)	7.4 (3.0)	–	0.023
Endoscopic Mayo 2	10	5	–	0.262
Endoscopic Mayo 3	1	3	–	0.262
Montreal classification extent^c^			1.0	–
E2 (%)	3 (27%)	3 (38%)	N/A	–
E3 (%)	8 (73%)	5 (63%)	N/A	–
Montreal classification severity^c^			0.55	–
S1 (%)	1 (9%)	2(25%)	–	–
S2 (%)	10(91%)	6(75%)	–	–
S3	0	0	–	–
S4	0	0	–	–
Median CRP mg/L (IQR)	2.50(11.5)	11.0(14.1)	N/A	0.09
Median calprotectin^a^ mg/kg (IQR)	904(872)	2313(1243)	N/A	0.037
Calprotectin <250 (n)	0	0	–	–
Calprotectin 250–1000 (n)	5	1	–	–
Calprotectin >1000 (n)	5	4	–	–
Calprotectin NA (n)	1	3	–	–
Median Nancy score^b^ (IQR)			N/A^c^	–
Inflamed biopsies	3(0.75)	2.5(1)	–	–
Uninflamed biopsies	0(0)	0(0)	–	0.45
Other medication at inclusion				
5-ASA (%)	8(73%)	8(100%)	1 (6.25%)	0.2281
GCS (%)	6(55%)	5(63%)	0 (0%)	1
Immunomodulator (%)	2(18%)	1(6%)	0 (0%)	0.6027
Cortiment (%)	3(27%)	0(0%)	0 (0%)	0.2281

IQR, interquartile range; SD, standard deviation; 5-ASA, 5-aminosalicylic acid; GCS, glucocorticosteroids.

a Within two weeks after initiating anti-TNF.

b Only the biopsies used for laser microdissection.

c Histological evaluation was available for 10/16 non-IBD controls without signs of inflammation.

Given that serum cytokine levels are typically elevated in IBD^15^ and serum is an ideal medium for biomarkers, we performed baseline serum cytokine multiplex analysis of anti-TNF responders and non-responders contrasted with non-IBD controls (*n* = 16). Cytokine concentrations ranged from <10 to >5,000 pg/mL (Figure 1B), and for most individual cytokines, median concentrations showed a progressive trend with controls having the lowest levels, non-responders’ intermediate levels, and responders the highest levels. *Z* score transformation confirmed this pattern (Figure 1C). Linear mixed-effects model (LMM) analysis revealed cytokine-specific response patterns (Figure S2C; Table S3); still, responders had an overall elevated cytokine profile with higher estimated marginal means compared to non-responders (Δ0.38, Padj = 0.055) (Figure 1D). Multivariate principal component analysis (PCA) (PC1: 36.2%, PC2: 12.2%) did not discriminate between response groups but revealed greater inter-individual variance in cytokine profiles of non-responders compared to responders (Figure S2D). PC1 composite scores were significantly elevated in both responders (Padj <0.0001) and non-responders (Padj = 0.029) vs*.* non-IBD controls, with a trend toward highest scores in responders compared to non-responders (Padj = 0.29) (Figure S2E). Partial least squares discriminant analysis (PLS-DA) further characterized these differences, confirming increased heterogeneity in non-responders (Figure S2F) and identified eight cytokines (IL12p70, IFNγ, IL1β, CCL11, IL9, IL17, IL4, and IL10) with variable importance in projection scores >1.0 that contributed to distinguishing responders from non-responders (Figure S2G).

### Inflammation elicits both distinct and shared gene signatures in colonic epithelium and lamina propria from patients with UC

**Figure 2:**
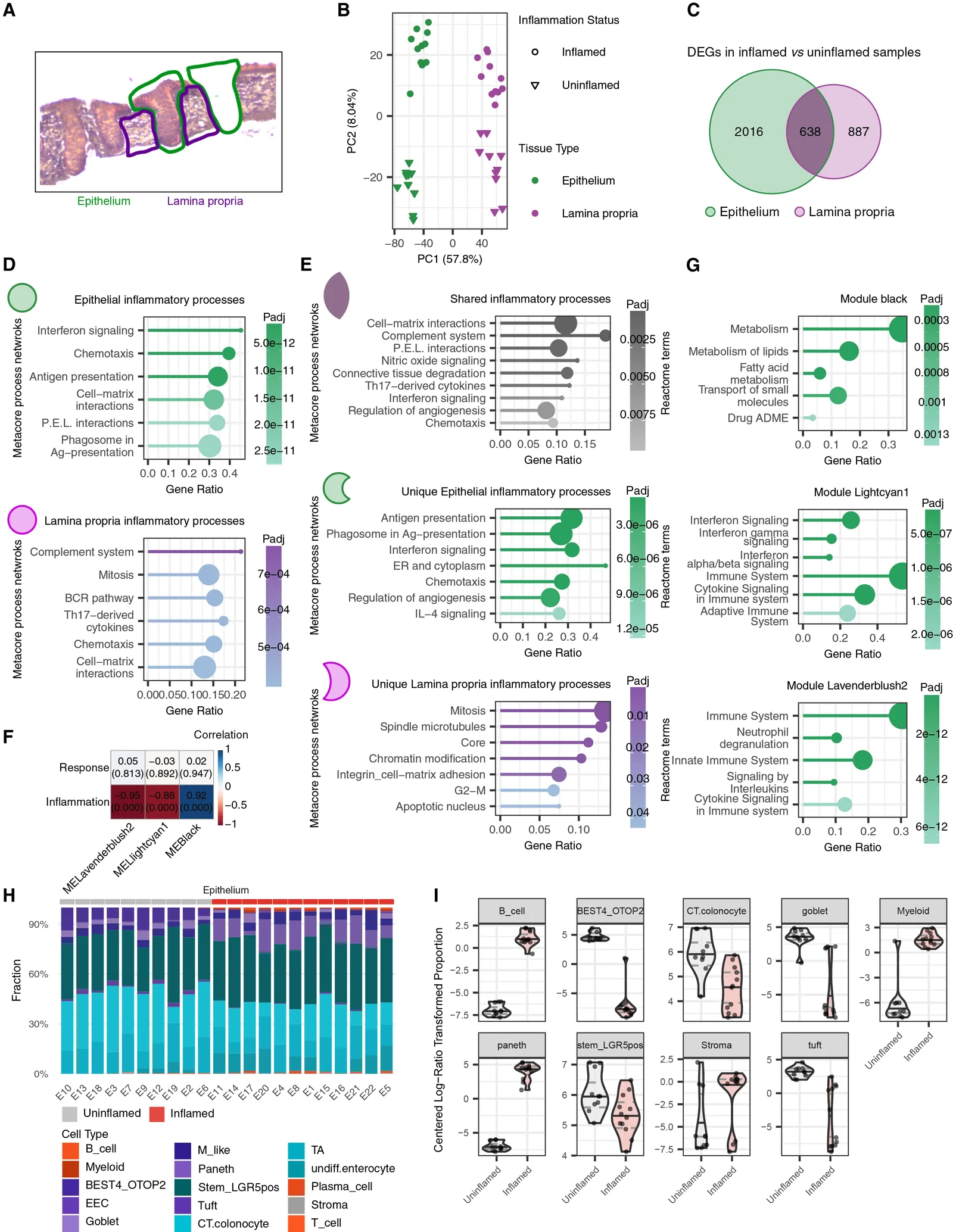
Mucosal compartment-specific transcriptional signatures and epithelial cell type analysis (A) Illustration of epithelial and lamina propria compartment outlining. Using the Leica LMD7 microscope, epithelial and lamina propria compartments were traced by hand and isolated in separate tubes with a laser. (B) Principal component analysis (PCA) of uninflamed and inflamed epithelial and lamina propria samples (*n* = 43). (C) Venn diagram of DEGs (Padj <0.05, log2FC > 0.3) between inflamed and uninflamed epithelium (*n* = 22) and lamina propria (*n* = 21). (D and E) Top enriched Metacore networks (Padj <0.01) for inflammation-upregulated (D) DEGs in each compartment or (E) compartment-unique/overlapping DEGs. P.E.L = Platelet-endothelial-leucocyte. (F) WGCNA modules correlating with inflammation (red) but not response status in epithelium. Each cell displays Pearson’s correlation coefficient with (Padj) (*n* = 22). (G) Top Reactome enrichment (Padj <0.01) for epithelial modules in FE. Gene ratio = DEGs vs*.* total number of genes; circle size = number of intersecting genes in each network. (H) Cell type composition in epithelial samples by deconvolution^10^ (*n* = 22). The cell type legend’s vertical orientation corresponds with the stacking order from top to bottom of cell populations in the plot. (I) Significantly different cell populations (FDR <0.05) between uninflamed and inflamed epithelium states (Centered Log-Ratio-transformed proportions). Also see Figures S3 and S5.

Although UC can be considered a systemic disease,^16^ serum cytokine measurements are confounded by non-intestinal inflammation. Given the complex and incompletely understood interplay of inflammatory processes in the epithelium and lamina propria, spatial characterization of molecular processes within the colonic mucosa remains essential. First, to characterize the compartment-specific transcriptional signatures during inflammation, we analyzed paired uninflamed (*n* = 10) and inflamed (*n* = 12) epithelium and uninflamed (*n* = 10) and inflamed (*n* = 11) lamina propria samples acquired before anti-TNF treatment. A visual representation of how epithelial and lamina propria compartments were delineated can be found in Figure 2A. The inflammation status was confirmed histologically by the Nancy score (Table 1). PCA displayed clear separation between tissue types (PC1) and inflammatory states (PC2), explaining 57.8% and 8.04% of the variance, respectively (Figure 2B). The epithelium showed greater inflammatory transcriptional activity in general, with 2,654 DEGs versus 1,525 in lamina propria (Table S4), while 638 DEGs were shared between the tissue types (Figure 2C). Enrichment analyses of compartment-specific DEGs (Figure 2D) revealed that inflamed epithelium was enriched for interferon signaling, chemotaxis, cell-matrix interactions, and antigen presentation, in contrast to complement processes, cell cycle, mitosis, B cell receptor signaling, IL17-derived cytokines, and chemotaxis in lamina propria. Common processes in the shared DEGs were complement system, Th17 responses, chemotaxis, and leukocyte recruitment (Figure 2E). Unique DEGs revealed antigen presentation and interferon response as epithelium-specific, while cell cycling processes were lamina propria-specific.

Weighted gene co-expression network analysis (WGCNA) identified several co-expressed gene modules representing biological processes in each compartment that correlated with inflammation status (Figures S3 and S4). Enrichment analysis of the epithelial gene modules (Figure 2F) showed that module Black was associated with uninflamed epithelium and enriched for fatty acid metabolism and peroxisome proliferator-activated receptor gamma (PPARγ) signaling (Figures 2G and S5A). Modules Lightcyan1 and Lavenderblush2 correlated with inflammation, with Lightcyan1 enriched for interferon signaling, viral infection, and antigen presentation, while Lavenderblush2 was enriched for TNF and IL-17 signaling and innate immune responses (Figures S5B and S5C). Cellular deconvolution using a colonic single-cell atlas (815,900 transcriptomes),^10^ confirmed that our epithelial compartment transcriptome was mainly related to epithelial cell types (Figure 2H). During inflammation, the inferred relative contribution from several epithelial subtypes (BEST4-OTOP2, Goblet, Tuft, Stem_LGR5pos, CT colonocytes) significantly decreased (Figure 2I), while B-, myeloid-, stromal- and Paneth cells increased. However, the relative contribution from B-, stromal-, and myeloid cells was low, which affects the robustness of the result.

**Figure 3:**
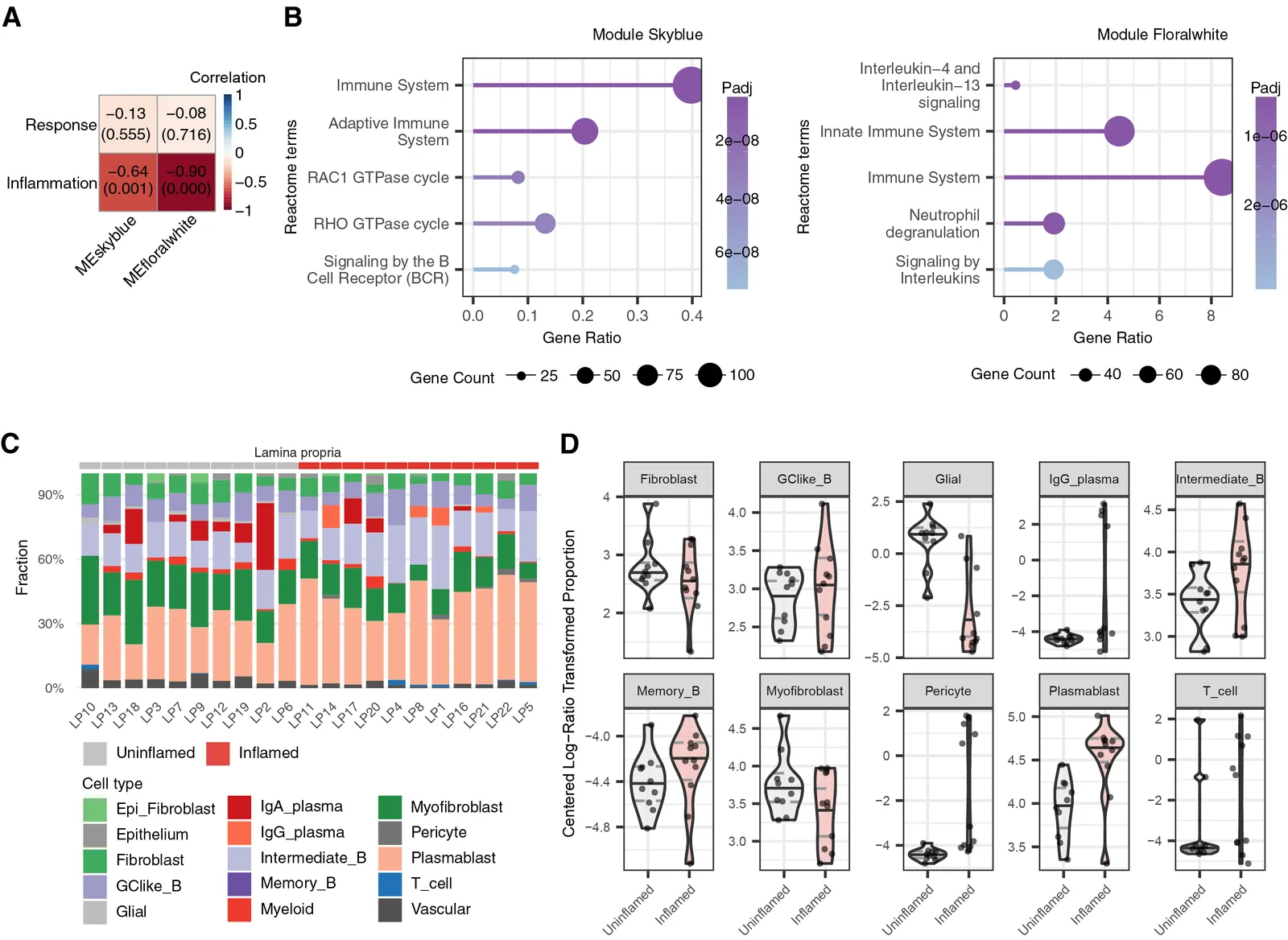
Lamina propria specific transcriptional network and cell type analysis (A) WGCNA modules correlating with inflammation (red) but not response status in lamina propria. Each cell displays Pearson’s correlation coefficient with (Padj) (*n* = 21). (B) Top Reactome enrichment (Padj <0.01) of modules in (A). Gene ratio = DEGs vs*.* total number of genes; circle size = number of intersecting genes in each network. (C) Cell-type composition in lamina propria samples by deconvolution (*n* = 21). The cell type legend’s vertical orientation corresponds with the stacking order from top to bottom of cell populations in the plot. (D) Significantly different cell populations (FDR<0.05) between uninflamed and inflamed lamina propria states (centered log-ratio-transformed proportions). Also, see Figures S4 and S5.

Enrichment analyses of the lamina propria gene modules showed that the modules Skyblue and Floralwhite (Figure 3A) correlating with inflammation were enriched for adaptive immune processes including B- and T cell receptor and NF-κB signaling pathways, or innate immune processes such as neutrophil degranulation and cytokine signaling (IL17, TNF, IL4, IL13), respectively (Figures 3B, S5D, and S5E). Consistent with this, cellular deconvolution showed that the lamina propria transcriptome was predominantly related to adaptive immune cells and fibroblasts (Figure 3C). The inferred relative contribution from germinal center (GC)-like, intermediate, and memory B cell and plasmablast signatures significantly increased during inflammation, while fibroblast, glial cell, and myofibroblast-associated signals decreased (Figure 3D). IgG plasma cell, pericyte, and T cell signatures showed a bimodal pattern during inflammation, with one patient cluster resembling uninflamed samples and another showing a noticeable increase.

### Anti-TNF non-responders show increased expression of cell cycle genes in lamina propria at baseline

Having delineated molecular processes in the lamina propria and epithelium contributing to mucosal inflammation, we next examined whether there were compartment-specific baseline molecular differences between anti-TNF non-responders and responders. Given the relevance of baseline expression of drug targets, we first examined regulation of the TNF pathway components depending on inflammation and response status using LMM analysis. *TNF* expression was elevated in uninflamed epithelium of non-responders but was markedly upregulated during inflammation in responders only, resulting in higher inflamed epithelial *TNF* levels in responders compared to non-responders, albeit with a bimodal distribution (Figure S6A) (Table S7). No significant *TNF* modulation was observed in the lamina propria by inflammation, response status, or their interaction. In keeping with their compartment-specific expression, *TNFRSF1A* was downregulated in inflamed lamina propria, and *TNFRSF1B* was upregulated in inflamed epithelium, with neither receptor differing between response groups.

**Figure 4:**
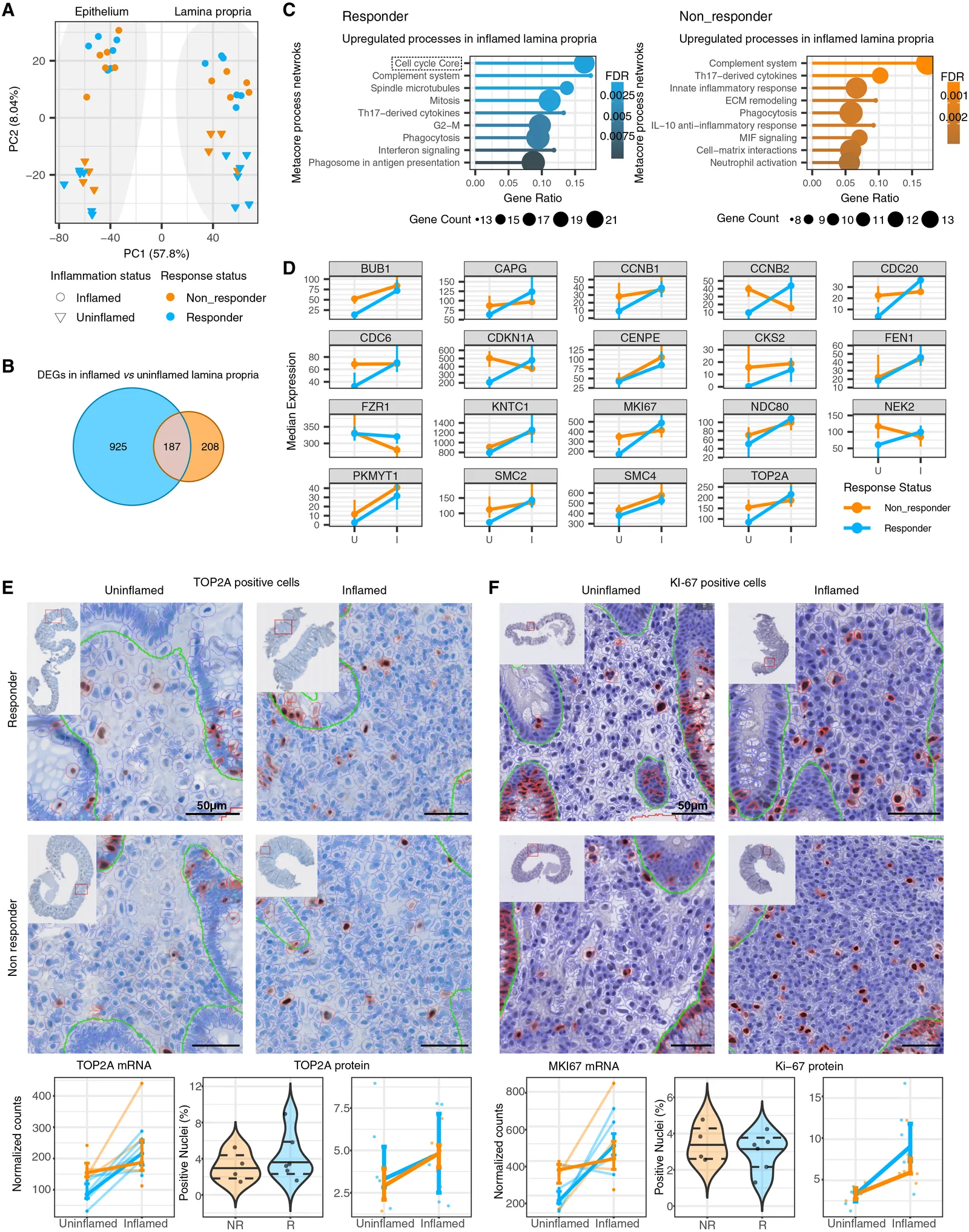
Baseline molecular differences in lamina propria between anti-TNF responders and non-responders (A) Principal component analysis (PCA) of uninflamed and inflamed epithelial and lamina propria samples (*n* = 43), colored by response status. (B) Venn diagram of DEGs (log2FC > 0.3, Padj <0.05) between inflamed and uninflamed lamina propria in responders (*n* = 12) and non-responders (*n* = 10). (C) Top enriched Metacore networks (Padj <0.01) for inflammation-upregulated DEGs. Gene ratio = DEGs vs*.* total number of genes; circle size = number of intersecting genes in each network. (D) Normalized expression of *Cell cycle core* genes in uninflamed (U) and inflamed (I) lamina propria in responders and non-responders (median ± interquartile range). (E and F) Representative images from computational quantification in the lamina propria of immunostained (E) TOP2A + or (F) KI67+ cells (red circles). The graphs show respective gene expressions and quantified proteins (median ± interquartile range) for responders (orange) and non-responders (blue). Violin plots show protein quantification within uninflamed lamina propria. Scale bars, 50 μm (E and F).

The PCA plot revealed overlap between the anti-TNF non-responders and responders in both inflamed epithelium and lamina propria. In the uninflamed compartments, a clustering tendency was observed, particularly in the lamina propria, where three of four non-responder samples grouped closer to the inflamed samples, suggesting a resemblance (Figure 4A). No DEGs were identified between the response groups in inflamed lamina propria nor in the uninflamed or inflamed epithelium. For uninflamed lamina propria, there were only 102 DEGs, including several mainly epithelial genes despite the generally good compartment separation, thus limiting further analysis (Figure S6B; Table S5). Since main differences were found in the lamina propria samples, separate inflammatory profiling of this compartment was done in non-responders and responders (Figure 4B), revealing both common and divergent transcriptional responses with 1,112 DEGs (925 unique) in responders, 395 DEGs (208 unique) in non-responders and 187 shared genes. While both groups exhibited Th17-associated processes, phagocytosis, and complement activation, cell cycle processes were uniquely enriched in responders during inflammation (Figure 4C). This was confirmed by plotting the *Cell cycle core* genes, showing higher expression in the uninflamed lamina propria of non-responders compared to responders, with diminished differences in inflamed tissue (Figure 4D). By immunohistochemistry and computational quantification, we verified that the number of TOP2A (Figure 4E) and KI67 (Figure 4F) positive cells increased during inflammation, although there was no obvious difference between the response groups in uninflamed tissue. Still, consistent with gene expression, the general proliferation marker KI67 tended to be higher in non-responders with less elevation in inflamed tissue, suggesting a somewhat altered proliferation pattern in the uninflamed lamina propria of non-responders.

### Uninflamed lamina propria of non-responders shows increased humoral activity compared to responders

**Figure 5:**
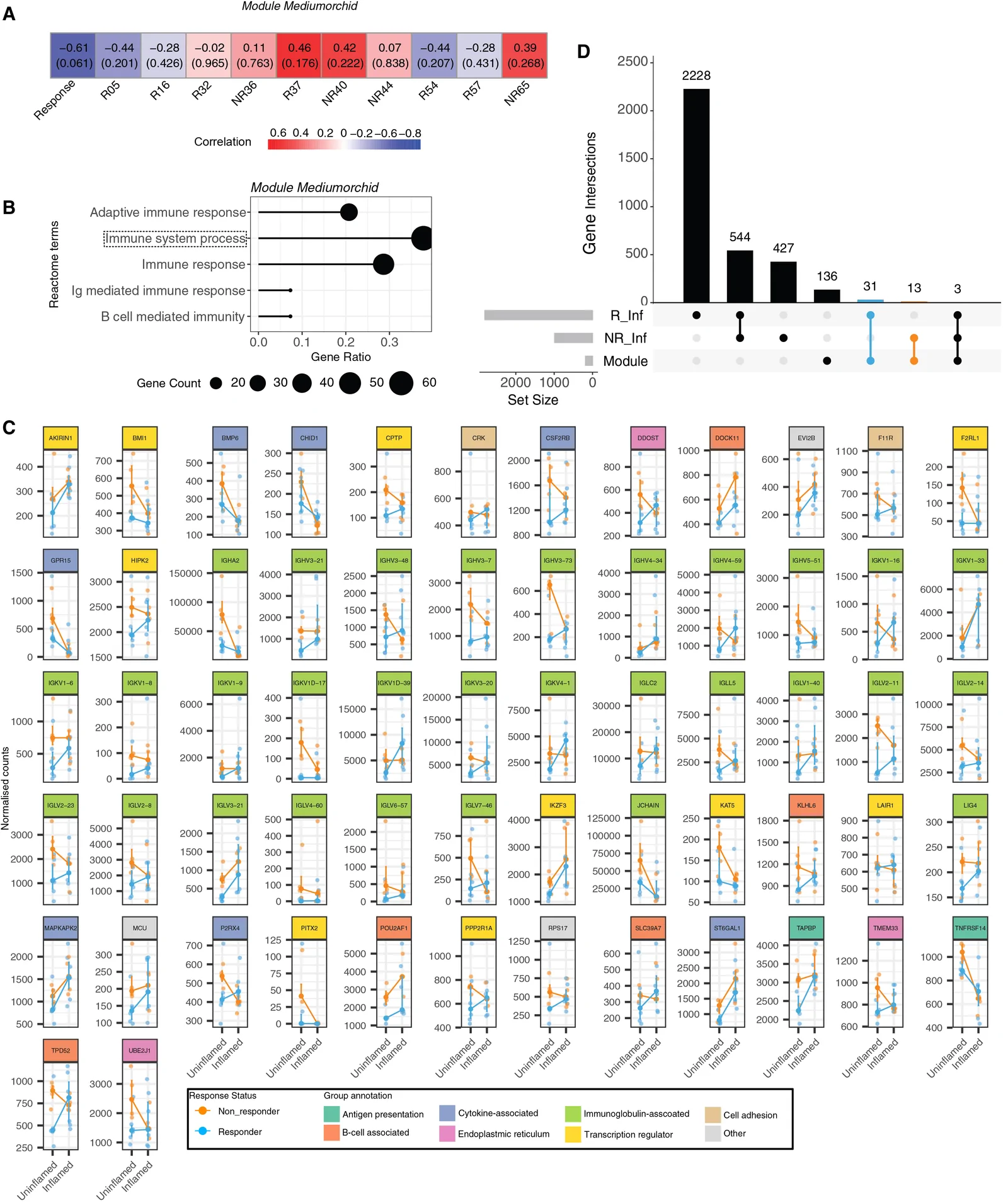
Module Mediumorchid reveals a humoral transcriptional signature in uninflamed lamina propria correlating with anti-TNF non-response (A) WGCNA module Mediumorchid in uninflamed lamina propria (*n* = 10) correlating with non-response status (blue) for responders (R) and non-responders (NR), showing Pearson’s correlation coefficient with Padj. (B) Top Reactome enrichment (Padj <0.00001) for module Mediumorchid. Gene ratio = DEGs vs*.* total number of genes; circle size = number of intersecting genes in each network. (C) Expression of *Immune system process* genes in lamina propria of responders and non-responders (median ± interquartile range). (D) Unique and overlapping lamina propria expression between Module Mediumorchid genes, inflamed vs*.* uninflamed DEGs in responders (R_inf) and inflamed vs*.* uninflamed DEGs in non-responders (NR_Inf). Also see Figure S9.

Given the differences in uninflamed lamina propria between the response groups, we sought to further identify the underlying biological processes by network analysis. WGCNA (Figure S7) revealed gene networks associated with response status, where module Skyblue1 showed increased viral translation and ribosomal ribonucleic acid activity correlating with non-response, while module Magenta correlated with response and was enriched in extracellular matrix organization (Figure S8). Module Mediumorchid correlated with non-responders (r = −0.61, Padj 0.061) and was enriched for adaptive immune system processes and B cell-mediated immunity (Figures 5A and 5B). The dominant Reactome term *Immune system process* comprised numerous immunoglobulin genes, including variable segments of heavy chains (*IGHV3-7, IGHV3-73, IGHV4-59, IGHV5-51*) and light chains kappa (*IGKV1-6, IGKV1-8, IGKV1D-17, IGKV3-20*) and lambda (*IGLV2-11, IGLV2-14, IGLV3-21, IGLV6-57*), representing antigen-binding variable domains (Figure 5C). Notable genes included *IGHV4-34* (Padj = 0.070; Figure S9), commonly elevated in autoimmune disorders,^17^ the IgA2 heavy chain constant region (*IGHA2*), and the joining chain (*JCHAIN*) essential for IgA and IgM multimerization and mucosal transport. Additional B cell-associated genes encompassed cytokine signaling (*GPR15, CHID1, CSF2RB*), transcriptional regulation (*BMI1, IKZF3, POU2AF1*), antigen presentation (*TAPBP*), and endoplasmic reticulum processes (*UBE2J1*). All genes demonstrated systematically higher expression in uninflamed lamina propria of non-responders vs*.* responders, with differences disappearing in inflamed tissue, similar to the *Cell cycle core* genes (Figure 4D). Module Mediumorchid also consisted of more and unique genes regulated during lamina propria inflammation in responders (*n* = 31) compared to non-responders (*n* = 13) (Figure 5D), implying elevated levels in the non-responders’ uninflamed tissue. By similar WGCNA analyses, we did not detect biologically meaningful co-expressed gene modules correlating with response status in either inflamed lamina propria or in uninflamed and inflamed epithelium. Although not statistically significant (Fisher’s exact test, *p* = 0.57), baseline corticosteroid use was numerically higher among non-responders (4/6) than responders (2/6) in the cohort selected for laser microdissection and RNA sequencing. Because corticosteroids can alter immune-cell transcriptomes, we assessed potential confounding and found that corticosteroid use did not affect the results (Figure S10).

**Figure 6:**
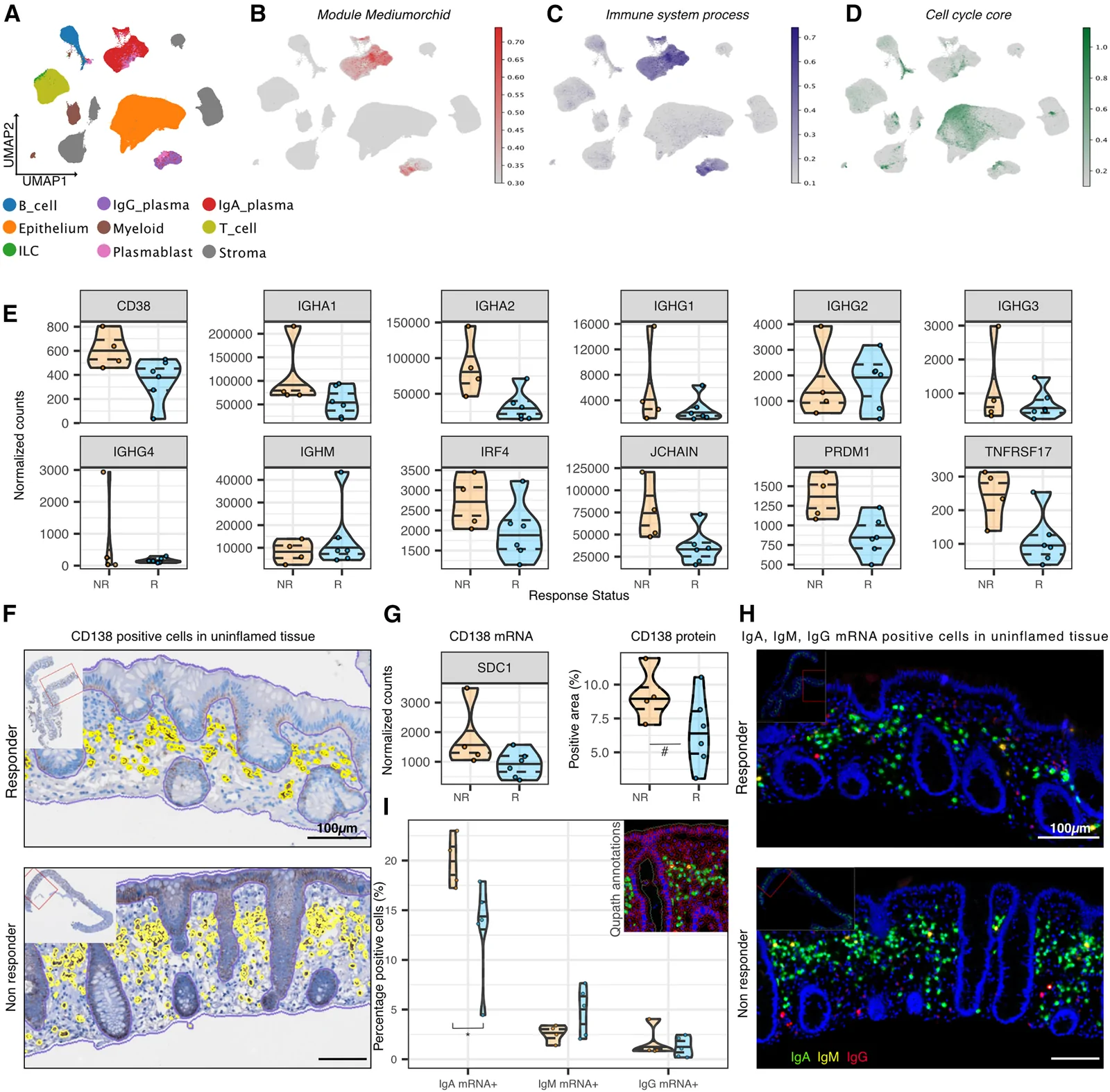
Module Mediumorchid defines a plasma cell-driven gene network associated with anti-TNF non-response (A–D) UMAP of colonic mucosal cells from single-cell RNA sequencing^10^ (*n* = 815,900 cells). (B and C) Single-cell expression of (B) module Mediumorchid, (C) *Immune system process,* and (D) *Cell cycle core* genes. (E) Gene expression of B- and plasma cell genes in uninflamed lamina propria of responders (R) and non-responders (NR) (median ± interquartile range). (F) Representative images from computational quantification of immunostained CD138 plasma cells (yellow) in uninflamed lamina propria of responders and non-responders (*n* = 10). (G) Graphs show respective gene expression and quantified protein (median ± interquartile range). # Mean NR vs. R difference in protein levels was 2.7 (95% Cl −0.72 to 6.2; *p* = 0.11, Welch’s *t* test). (H) Representative images from computational quantification of fluorescence multiplex *in situ* hybridization of mRNA for IgA (green), IgM (yellow), and IgG (red)-positive plasma cells in uninflamed lamina propria of non-responders and responders (*n* = 9). (I) Graphs show the percentage of positive cells in each channel relative to the total number of detected cells (DAPI-positive). Image shows example of QuPath annotations. ∗ Mean NR vs. R difference in number of IgA-positive plasma cells was 6.7 (95% Cl 0.19 to 13; *p* = 0.045, Welch’s *t* test), while IgM- and IgG-plasma cells were not significantly different. Scale bars, 100 μm (F and H).

To elucidate which cell types may underlie the identified gene networks in the uninflamed lamina propria of non-responders, single-cell projection using the Thomas et al.^10^ atlas (Figure 6A) demonstrated that the module Mediumorchid genes (Figure 6B) and *Immune system process* genes (Figure 6C) were predominantly expressed in plasma cell populations, particularly plasmablasts and IgA but also IgG plasma cell subsets. *Cell cycle core* genes (Figure 6D) were also highly expressed in B cell subsets and IgA and IgG plasmablasts. Despite not finding significant differences in inferred relative cellular abundance between the response groups via cellular deconvolution, multiple B- and plasma cell-associated genes showed elevated expression in uninflamed lamina propria of non-responders, particularly pointing to increased IgA and not IgG subsets (Figure 6E). This was verified *in vivo* using tissue staining and computational quantification. Immunostaining showed generally more CD138-positive plasma cells in the uninflamed lamina propria of non-responders compared to responders (Figures 6F and 6G). This was further characterized using multiplex *in situ* mRNA hybridization, which confirmed that the higher plasma cell load in non-responders was due to increased IgA mRNA-positive subtypes, but not IgM- and IgG mRNA-positive subtypes (Figures 6H and 6I), all in agreement with the gene expression (Figure 6E).

**Figure 7:**
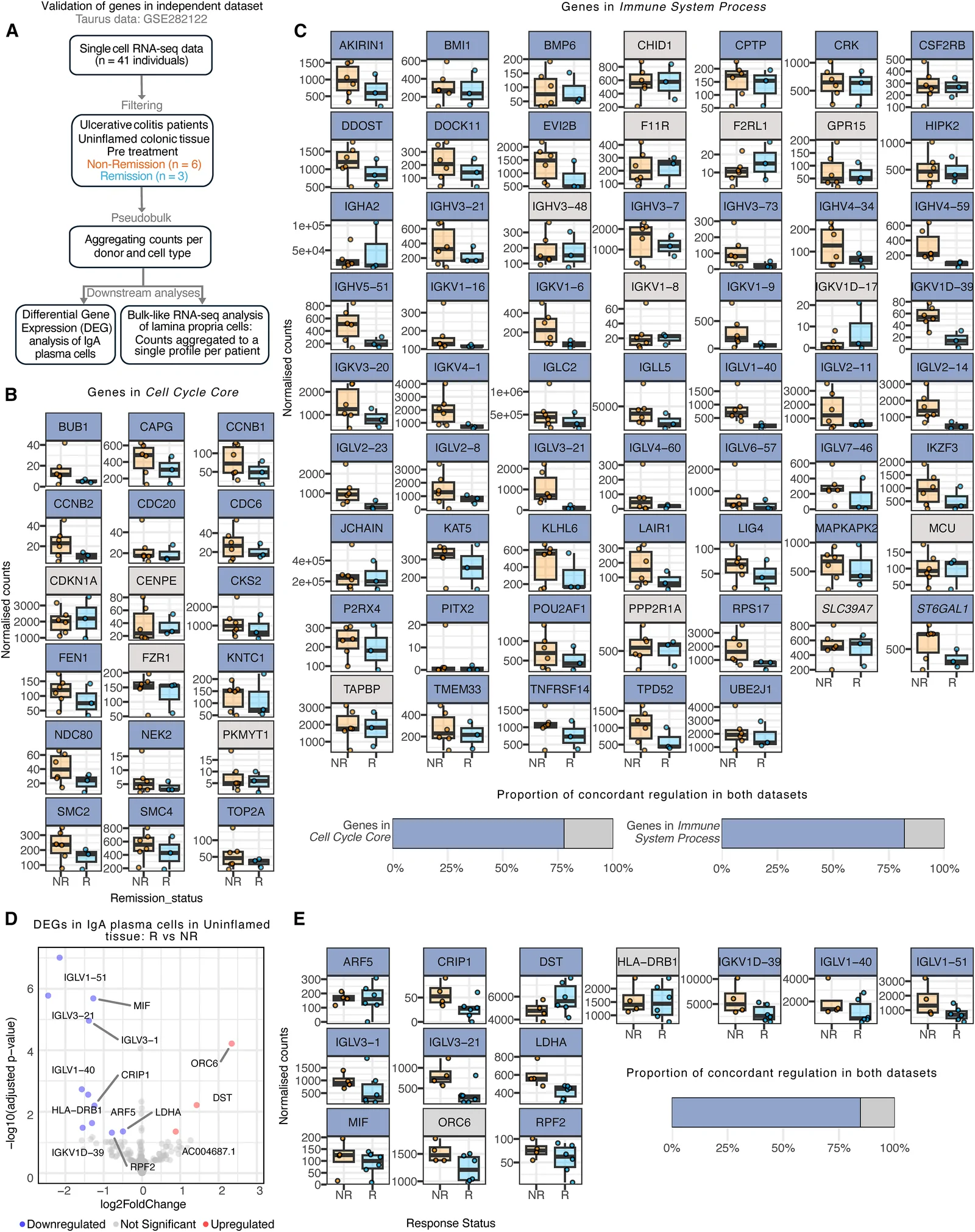
Validation in an independent cohort of cell cycle and humoral gene signatures in anti-TNF non-responder uninflamed lamina propria (A) Flow-chart of validation in an independent single-cell RNA sequencing dataset using cell filtering and generation of individual pseudobulk profiles (*n* = 9) for comparison to our data (*n* = 10). (B and C) Expression in pre-treatment uninflamed lamina propria cells in non-remission (NR) and remission (R) patients in the validation cohort of (B) *Cell cycle core* and (C) *Immune system process* genes (median ± interquartile range). (D and E) DEGs (log2FC > 0.3, Padj <0.05) between remission (R) and non-remission (NR) IgA plasma cells in the validation cohort and (E) expression in uninflamed lamina propria of responders (R) and non-responders (NR) in our dataset. Blue labels and graphs indicate concordant regulation (%) in both datasets.

To further validate the identified gene pattern in uninflamed lamina associated with anti-TNF non-response, we performed pseudobulk analyses using data from pre-treatment uninflamed lamina propria cells from 9 UC in the independent single-cell anti-TNF treatment cohort from Thomas et al.^10^ (Figure 7A). Both datasets showed concordant increased regulation in non-responders compared to responders in 78% (14 of 18) of the *Cell cycle core* genes (Figure 7B) and 82% (50 of 61) of the *Immune system process* genes (Figure 7C). Lastly, their single-cell data revealed DEGs between uninflamed IgA plasma cells of non-remission and remission (Figure 7D; Table S6), which had 85% (11 of 13) concordant regulation in uninflamed lamina propria in our data (Figure 7E).

## Discussion

Our mucosal compartment-specific transcriptomic analysis of paired uninflamed and inflamed colonic mucosa reveals a humoral immune signature in uninflamed lamina propria of anti-TNF non-responders at baseline, characterized by elevated expression of cell cycle, immunoglobulin, and plasma cell-associated transcriptional programs. These findings address the critical clinical challenge of unpredictable anti-TNF therapeutic response^7^ and reveal that even histologically uninflamed tissue can carry fundamental differences in baseline immune architecture that may represent an underlying predisposition to non-response.

Humoral activity in uninflamed colonic mucosa was also described by Uzzan et al.,^18^ with increased prevalence of IgG plasma cells and switched memory B-cells compared to healthy controls, suggested to reflect past inflammation or dissemination of inflamed cells. Although the exact pre-inflammation status of the uninflamed tissue in our non-responders compared to responders is impossible to know for certain, no differences in earlier pan-colitis are indicated (Figure S1B). Also, while Uzzan et al.^18^ compared to healthy controls, we differentiated response groups that both had active disease and found alterations in IgA cells and germline immunoglobulin genes which may be more indicative of inherent variations. The immunoglobulin-enriched signature may suggest autoinflammatory mechanisms. Particularly, IGHV4 (often *IGHV4-34*) genes are associated with intrinsic autoreactivity^19^ and present in multiple autoimmune disorders,^17^ such as rheumatoid arthritis associated with UC,^20^ and Crohn’s disease.^17^ Although we did not measure or characterize autoreactive antibodies directly, the high therapeutic s-drug concentrations measured make anti-drug antibodies unlikely as the underlying mechanism for non-response. Still, non-responders seemed to need higher dosage and shorter intervals of infliximab and had somewhat lower adalimumab levels, which could mean that the increased humoral activity somehow interacts and affects the anti-TNF antibodies without giving sub-clinical s-trough levels. This hypothesis is based on only a few observations and should be interpreted with caution. Primary anti-TNF non-responders are reported less likely to respond to second-line biologics^21^ and may benefit from alternative drugs (such as small-molecule JAK-inhibitors). Although looking at both uninflamed and inflamed tissues, the converge with other studies in UC^9^^,^^13^ and Crohn’s disease^22^ strengthens evidence for humoral involvement in anti-TNF resistance across IBD phenotypes.

Successful microdissection enabled distinct profiling of epithelial and lamina propria compartments across inflammatory states, showing enhanced processes in both inflamed epithelium^10^^,^^23^ and lamina propria^10^ consistent with previous studies. Aligning with our results, Uzzan et al.^18^ demonstrated expansion of plasmablast and IgG+ plasma cells during UC inflammation. Interestingly, in our cohort there was a bimodal distribution of IgG plasma cells in inflamed tissue, either maintaining uninflamed-like abundance or showing marked expansion, suggesting distinct inflammatory endotypes. Uzzan et al.^18^ also reported humoral antibodies with reduced VDJ gene mutations and diversity, which is associated with extrafollicular development predisposing to expansion of autoreactive B cell clones.^24^^,^^25^ They pointed to IFNγ as a potential driver of the dysregulated humoral response, consistent with our enrichment of type II interferon responses during mucosal inflammation. The IBD-specific histological feature basal plasmacytosis is typical at early stages and can be an independent indicator of relapse.^26^ Further support comes from circulating autoantibodies against perinuclear anti-neutrophil cytoplasmic antibodies (pANCA)^27^ and the newly identified autoreactive plasmablast clones against αvβ6,^18^ which interestingly is associated with aggressive disease course^28^ and is detectable in the pre-clinical stage, suggesting early involvement of dysregulated humoral responses.^29^^,^^30^ Importantly, the recent report^31^ of rapid and complete drug-free remission in a severe multidrug-resistant UC patient by CD19 chimeric antigen receptor T cell (CAR-T) therapy aligns with our findings and strongly suggests involvement of the B-cell lineage in the pathogenesis of at least a subpopulation of patients with UC.

Contrary, in serum there was a tendency toward higher median cytokine concentrations in anti-TNF responders compared to non-responders, including for central UC-related cytokines such as IL17, TNF, CCL11, CXCL10, and IFNγ.^23^^,^^32^ We also observed increased inter-individual heterogeneity among anti-TNF non-responders, which could indicate less systemic and coordinated TNF-driven disease in this group. Although analyses did not indicate differential expression of TNF and its receptors as a basis for varying anti-TNF responses, epithelial *TNF* expression was higher and more triggered by inflammation in responders compared to non-responders. Increased membrane-bound TNF expression has similarly been demonstrated to correlate with both short- and long-term response,^33^ and previous serum proteomic profiling of 460 proteins from 153 patients with UC found that 15 proteins representing TNF-independent pathways predicted treatment escalation.^16^ Others have found incongruent regulation of mucosal TNF and its receptors between response groups.^6^^,^^34^ Thus, the complex anti-TNF mechanisms remain incompletely understood. Nevertheless, our observations fit with the concept that anti-TNF response is driven by other molecular inflammatory mechanisms than TNF. However, serum analyses are potentially influenced by several systemic factors and are just a proxy for the colonic mucosal environment, from which a more direct and nuanced understanding of response heterogeneity could be revealed.

Key strengths of this study include our stringent inclusion criteria, separation of the epithelium and lamina propria mucosal compartments, and paired uninflamed and inflamed biopsies, ensuring homogenous and pure datasets and control of individual variability. Notably, the findings were validated through multiple methodologies, including recent data from an independent, large-scale longitudinal therapeutic single-cell RNA-sequencing atlas. The lack of transcriptomic differences in inflamed tissue between response groups, as for the previously reported OSM expression,^11^ may reflect study design and our extended 6-month endoscopic assessment. The timing aligns with Friedrich et al.'s^13^ approach but differs from STRIDE-2 recommendations^35^ and older studies^4^^,^^11^^,^^12^^,^^36^ assessing primary non-response at earlier timepoints. While our 6-month endpoint captures sustained response, it may arguably conflate primary mechanistic non-response with secondary loss of response. Furthermore, whether the identified uninflamed humoral signature actively interferes with anti-TNF therapy or simply acts as a proxy for a more aggressive, entrenched disease phenotype that is generally harder to treat, remains to be determined. Our study has a relatively small sample size, and larger cohorts will be necessary to confirm and extend our findings. The study was, however, designed with a strong emphasis on patient homogeneity and paired compartment-specific sampling, thereby reducing biological and clinical variability and strengthening internal validity. Notably, the main finding of a distinct humoral and cell cycle signature in uninflamed lamina propria of anti-TNF non-responders was confirmed in the separate dataset, also with a limited sample size.

There are probably several distinct UC endotypes and mechanisms for anti-TNF non-response involving TNF-independent inflammatory pathways. Our mechanistic framework identifies inherent humoral activation in uninflamed colonic mucosa as a potential driver of anti-TNF resistance in UC, with clinical implications.

### Limitations of the study

Missing samples in non-responders obscured whether fecal calprotectin differed between groups, but the clinical utility of calprotectin as a predictor of anti-TNF response is debated.^37^ Baseline medication, particularly corticosteroids, is a potential confounder of the mucosal transcriptome. Although a higher proportion of non-responders were receiving corticosteroids, this difference was not significant, and complementary analyses indicated that the observed humoral signature reflects non-response rather than a glucocorticoid effect (Table S8). Residual confounding cannot be fully excluded given the limited sample size. Furthermore, our study was underpowered for detecting smaller effect sizes, particularly for subgroup analyses, giving only notable trends for some of the results.

## Resource availability

### Lead contact

Further information and requests for resources should be directed to and will be fulfilled by the lead contact, Ingunn Bakke (ingunn.bakke@ntnu.no).

### Materials availability

This study did not generate new unique reagents. Remaining human biological materials (FFPE biopsies and serum) are limited and were collected under informed consent; requests for access will be considered by the lead contact, subject to ethical and legal restrictions and a material transfer agreement.

### Data and code availability

• Bulk RNA sequencing data have been deposited at GEO and are publicly available as of the date of publication. The accession number (GEO: GSE288517) is listed in the key resources table.
• All original code has been deposited at Zenodo and is publicly available as of the date of publication. The DOI (https://doi.org/10.5281/zenodo.17593185) is listed in the key resources table.
• Any additional information required to reanalyze the data reported in this paper is available from the lead contact upon request.

## Acknowledgments

This work was performed in collaboration with the Gastrointestinal Endoscopy Unit at the Department of Gastroenterology and Hepatology, St. Olav’s University Hospital, and could not be done without the help of dedicated nurses and doctors following the patients. We thank engineers Linn Karina M. Selvik, Siri Sæterstad, Liv Ryan, and Ann-Therese Chattergoon Ali, and MScPharm student Johan Lorentz Steffensen at the Department of Clinical and Molecular Medicine, Norwegian University of Science and Technology (NTNU) for their technical assistance, gastrointestinal pathologist Elin Synnøve Røyset at the Department of Pathology, St. Olav’s University Hospital, for the histological evaluation, Vidar Beisvåg and Tone Christensen at the Genomics Core Facility (GCF), NTNU, for valuable support, and associate professor Turid Follestad at the 10.13039/100009123Department of Public Health and Nursing, NTNU, for advising the cytokine linear mixed model. RNA library preparation, sequencing, and parts of the bioinformatics analysis were performed in close collaboration with the GCF. We thank the Cellular and Molecular Imaging Core Facility (CMIC), 10.13039/100009123NTNU, and the Department of Pathology, St. Olav’s University Hospital, for scanning the immunostained sections. Both GCF and CMIC are funded by the Faculty of Medicine and Health Sciences at NTNU, and the 10.13039/501100004590Central Norway Regional Health Authority. The study was funded by the 10.13039/501100004787Research Council of Norway (grant number FRIPRO 335204), the Liaison Committee between the 10.13039/501100004590Central Norway Regional Health Authority and 10.13039/100009123NTNU; and the Liaison Committee between St. Olav’s University Hospital and 10.13039/100019902Faculty of Medicine and Health Sciences at NTNU. The work was performed within the Clinical Academic Group for Precision Medicine in inflammatory bowel disease (CAG-IBD https://www.ntnu.edu/cag-ibd/), which is supported by the Liaison Committee for Education, Research and Innovation in Central Norway (project no. 90545800). Some of the data were presented as an oral Late-breaking session at UEGW in Berlin 2025.

## Author contributions

Conceptualization: G.A.E.W., A.E.Ø., A.K.S., T.B., and I.B. Methodology: G.A.E.W., S.S., L.F.K., A.vB.G., A.K.S., A.E.Ø., T.B., and I.B. Software: G.A.E.W., H.S.P., and A.F. Validation: S.S., H.S.P., and I.B. Formal analysis: G.A.E.W., S.S., L.F.K., A.S., H.S.P., A.F., A.vB.G., A.K.S., T.B., and I.B. investigation: G.A.E.W., S.S., L.F.K., K.L., H.F., A.E.Ø., A.K.S., T.B., and I.B. Resources: K.L., H.F., A.E.Ø., and A.K.S. Data curation: G.A.E.W., H.F., K.L., and A.K.S. Writing – original draft: G.A.E.W., S.S., T.B., and I.B. Writing – review and editing: All authors. Visualization: G.A.E.W. and S.S. Supervision: A.E.Ø., A.K.S., T.B., and I.B. Project administration: A.E.Ø., A.K.S., T.B., and I.B. Funding acquisition A.E.Ø., A.K.S., T.B., and I.B.

## Declaration of interests

The authors declare no competing interests.

## Declaration of generative AI and AI-assisted technologies in the writing process

During the preparation of this work, the authors used Claude.ai (Anthropic, San Francisco, CA, USA) to edit the text since English is not the authors' native language and to assist with writing R syntax. After using these tools or services, the authors reviewed and edited the content as needed and take full responsibility for the content of the publication.

## STAR★Methods

### Key resources table

**Table:** 

REAGENT or RESOURCE	SOURCE	IDENTIFIER
**Antibodies**		

Mouse monoclonal anti-Ki67 (clone MIB-1)	Dako/Agilent	Cat#M7240; RRID:AB_2142367
Rabbit polyclonal anti-TOP2A	Novus Biologicals	Cat#NBP1-85928; RRID:AB_11043073
Mouse monoclonal anti-CD138/Syndecan-1 (clone MI15)	Dako/Agilent	Cat#M7228; RRID:AB_2254116
EnVision-HRP/DAB+ secondary detection (REAL EnVision Detection System)	Dako/Agilent	Cat#K5007; Cat#K500711-2; RRID:AB_2888627

**Biological samples**		

Human colonic FFPE biopsies (uninflamed and inflamed mucosa), UC patients	This study; St. Olav’s University Hospital, Trondheim	N/A
Human serum (UC patients and non-IBD controls)	This study; St. Olav’s University Hospital, Trondheim	N/A

**Critical commercial assays**		

Bio-Plex Pro Human Cytokine 27-plex Assay	Bio-Rad Laboratories	Cat#M500KCAF0Y; RRID: AB_2893118
RNeasy FFPE Kit	Qiagen	Cat#73504
Qubit RNA HS Assay Kit	Thermo Fisher Scientific	Cat#Q32855
RNA 6000 Pico reagents and chips	Agilent Technologies	Cat#5067–1513
RNA 6000 Pico ladder	Agilent Technologies	Cat#5067–1515
Illumina Stranded Total RNA Prep with Ribo-Zero	Illumina	Cat#20040529
RNA UD Indexes Set C	Illumina	Cat#20091659
Collibri Library Quantification Kit	Thermo Fisher Scientific	Cat#A38524500
RNAscope Multiplex Fluorescent Reagent Kit v2	Bio-Techne	Cat#323135, 323100; RRID:SCR_018352
TSA Vivid Fluorophore 520	Bio-Techne	Cat#323271
TSA Vivid Fluorophore 570	Bio-Techne	Cat#323272
TSA Vivid Fluorophore 650	Bio-Techne	Cat#323273

**Chemicals, peptides, and recombinant proteins**		

PKD buffer (tissue digestion)	Qiagen	Cat#73505
Mag-Bind TotalPure NGS (library purification)	Omega Biotek	Cat#M1378-01
RNaseZAP (decontamination)	Sigma-Aldrich	Cat#R2020

**Deposited data**		

Bulk RNA sequencing data (this study)	This study; Gene Expression Omnibus (GEO)	GEO: GSE288517
Original/statistical analysis code	This study; Zenodo	https://doi.org/10.5281/zenodo.17593185
Single-cell RNA-sequencing colonic atlas (reference for deconvolution and validation)	Thomas et al.^10^	GEO: GSE282122

**Oligonucleotides**		

RNAscope Probe - Hs-IgGpan	Bio-Techne	Cat#439501
RNAscope Probe - Hs-IGHMC2	Bio-Techne	Cat#481091-C2
RNAscope Probe - Hs-IGHA1-A2-C3	Bio-Techne	Cat#1327221-C3

**Software and algorithms**		

R (v4.4)	R Core Team^38^	https://www.r-project.org/
ggplot2 (v4.0.3)	Wickham et al.^39^	https://ggplot2.tidyverse.org/
Ggridges (v0.5.7)	Wilke et al.^40^	https://wilkelab.org/ggridges/
Patchwork (v1.3.0)	Pedersen et al.^41^	https://patchwork.data-imaginist.com/
lme4 (v1.1-37)	Bates et al.^42^	https://cran.r-project.org/package=lme4
emmeans (v1.11.1)	Lenth^43^	https://cran.r-project.org/package=emmeans
DHARMa (residual diagnostics)	Hartig et al.^44^	https://cran.r-project.org/package=DHARMa
FactoMineR (v2.8)	Lê et al.^45^	https://cran.r-project.org/package=FactoMineR
factoextra (v1.0.7)	Kassambara & Mundt^46^	https://cran.r-project.org/package=factoextra
mixOmics (v6.26)	Rohart et al.^47^	https://www.bioconductor.org/packages/mixOmics
bcl2fastq (v2.2)	Illumina	https://support.illumina.com/
FastQC (v0.11.9)	Babraham Bioinformatics	https://www.bioinformatics.babraham.ac.uk/projects/fastqc/
fastp (v0.20.1)	Chen et al.^48^	https://github.com/OpenGene/fastp
STAR (v2.7.9a; aligner, QC)	Dobin et al.^49^	https://github.com/alexdobin/STAR
Picard (v2.21.5; CollectRNASeqMetrics)	Broad Institute	https://broadinstitute.github.io/picard/
Salmon (v1.7.0)	Patro et al.^50^	https://github.com/COMBINE-lab/salmon
tximport (v1.20.0)	Soneson et al.^51^	https://bioconductor.org/packages/tximport
DESeq2 (v3.21.0)	Love et al.^52^	https://bioconductor.org/packages/DESeq2
DAVID	Huang et al.^53^^,^^54^	https://davidbioinformatics.nih.gov/
MetaCore	Clarivate Plc	https://portal.genego.com/
gprofiler2 (v0.7.0)	Kolberg et al.^55^	https://cran.r-project.org/package=gprofiler2
WGCNA (v1.73)	Langfelder & Horvath^56^	https://cran.r-project.org/package=WGCNA
CIBERSORTx (Docker)	Newman et al.^57^^,^^58^	https://cibersortx.stanford.edu/
QuPath (v0.5.1)	Bankhead et al.^59^	https://qupath.github.io/
DeepLabV3+ tissue segmentation model (ResNet-18 encoder)	Pettersen et al.^60^; Røyset et al.^61^	See references
GraphPad Prism (v10.6.1)	GraphPad Software	https://www.graphpad.com/

**Other**		

Bio-Plex 200 System	Bio-Rad Laboratories	N/A
Leica LMD7 laser microdissection microscope	Leica Microsystems	N/A
2.0 μm PEN-membrane glass slides (RNase-free)	Leica Microsystems	Cat#11505189
Agilent 2100 Bioanalyzer	Agilent Technologies	N/A
Agilent 4150 TapeStation System	Agilent Technologies	Cat#G2991BA
Qubit Fluorometer	Thermo Fisher Scientific	N/A
Illumina NovaSeq 6000 Sequencing System (S2 flow cell; 59–10-10–59 bp)	Illumina	Cat#20028316
NanoZoomer S360 slide scanner	Hamamatsu Photonics	N/A
Olympus VS200 slide scanner	Evident/Olympus	N/A

### Experimental model and study participant details

#### Ethics

The study was approved by the Central Norway Regional Committee for Medical and Health Research Ethics, Norway (#26798). All participants provided written informed consent.

#### Human participants and study cohort

Biologically naive UC patients were prospectively recruited at initiation of anti-TNF therapy at St. Olav’s University Hospital, Trondheim, from 2018 to 2022. Before treatment (baseline, T0), patients underwent colonoscopy with biopsies, clinical and endoscopic Mayo scoring (MES), and serum collection (Figure 1A); assessments were repeated after 6 months (T1). Patients received either infliximab (5–10 mg/kg) or adalimumab (160–80–40 mg), with serum trough concentrations (s-trough) measured after 14 weeks for dose adjustment. Inclusion criteria for the present study were MES 2–3 at T0; Montreal E2–E3 (left-sided or extensive colitis); and a completed endoscopic evaluation at T1. Primary response (R) was defined as MES 0–1 at T1 (*n* = 11) and non-response (NR) as maintained MES 2–3 (*n* = 8) (Figures S1B and S1C). Non-IBD controls (*n* = 16) for the serum analyses were outpatients assessed without macroscopic or microscopic disease.

Participant sex, age, and additional demographic and clinical characteristics for each group, together with the sample sizes used in each analysis, are reported in Table 1. Ancestry, race, ethnicity, and socioeconomic status were not recorded at inclusion. The cohort was drawn from a single hospital catchment area in Trondheim, central Norway, a population that is predominantly of Northern European ancestry and that, under universal public health-care coverage, exhibits comparatively limited socioeconomic disparity; the cohort is therefore expected to be relatively homogeneous with respect to these variables, although this was not formally assessed. This limited variation, together with the modest sample size, constrains the generalizability of the findings to more ancestrally and socioeconomically diverse populations. The influence of sex on the results was not analyzed owing to the small sample size. This was a prospective cohort study, and no clinical trial number is applicable.

### Method details

An experimental overview is presented in Figure S1A. Detailed specifications for all experiments, computational tools, and analytical parameters, together with the multiplex and downstream RNA-sequencing data, are organized in Tables S1, S2, S3, S4, S5, S6, S7, S8, and S9.

#### Serum collection and multiplex assay

Serum was collected in separator tubes at T0 from the 18 included patients (one sample missed due to hospitalization) and 16 controls. After 20 min of clotting at room temperature (RT), samples were centrifuged (3000 rpm, 10 min, 4°C) and stored at −80°C. Twenty-seven cytokines were analyzed using the Bio-Plex Pro Human Cytokine 27-plex Assay on a Bio-Plex 200 System following the manufacturer’s protocol (1:4 dilution). Cytokines with more than 25% missing values were excluded (GM-CSF, IL-2, IL-5, IL-6, IL-15, VEGF, FGF-basic) (Table S1). Values below the detection limit were imputed at 50% of the lowest detectable value per cytokine. No values exceeded the detection limit.

#### Laser microdissection

Six responders and six non-responders were selected on the basis of optimal transverse orientation of the T0 biopsy. Hematoxylin & eosin-stained sections were scored with the Nancy histological index by a gastrointestinal pathologist. Paired formalin-fixed paraffin-embedded (FFPE) biopsies from uninflamed (right flexure) and inflamed (left colon) mucosa were used for each patient, except for two non-responders for whom only inflamed biopsies were available. FFPE sections (8 μm) mounted on 2.0 μm RNase-free glass PEN-membrane slides were heated (30 min, 60°C), hydrated, hematoxylin-stained (1 s), then dehydrated (graded alcohols and xylene) and desiccated (30 min) before microdissection with a Leica LMD7 microscope (5*x* objective). The microdissection process included delineating the epithelial and lamina propria compartments by hand using a PEN Screen before laser cutting and isolating the samples (Figure 2A). Laser cutting parameters were: power 30, aperture 3, speed 5, middle pulse count 3, final pulse 0, head current 1, and pulse frequency 1400.

Epithelium and lamina propria were collected into separate tubes (mean dissected areas per biopsy: epithelium 862,3565 μm^2^ and lamina propria 951,3853 μm^2^), supplemented with 150 μL PKD digestion buffer, vortexed, and stored at −80°C until RNA sequencing within 3 months. The final sample groups were uninflamed/inflamed epithelium and lamina propria (*n* = 6/6) from responders and uninflamed/inflamed epithelium and lamina propria (*n* = 4/6) from non-responders.

#### RNA isolation and bulk RNA sequencing

Total RNA was extracted using the RNeasy FFPE Kit per the manufacturer’s instructions, with a 10-min room temperature incubation before elution. RNA was quantified using the Qubit RNA HS Assay Kit, and integrity was assessed with the Agilent RNA 6000 Pico Kit on a 2100 Bioanalyzer. RNA sequencing libraries were prepared using Illumina Stranded Total RNA Prep with the Ribo-Zero kit, using all available RNA (range 6–55 ng). Following rRNA depletion, RNA was fragmented (94°C, 2 min) and reverse transcribed with random hexamers and an Actinomycin D-supplemented First Strand Synthesis Mix. Second-strand synthesis incorporated dUTP for strand specificity. After 3′ adenylation and adapter ligation, libraries were PCR-amplified (15 cycles) using Illumina RNA UD Indexes Set C. Libraries were purified, quantified, and validated, confirming 200–500 bp fragments (peak ∼280 bp). Pooled libraries were sequenced on a NovaSeq 6000 S2 flow cell. FASTQ files were generated using bcl2fastq.

#### Immunohistochemistry

FFPE sections (4 μm) were deparaffinized, quenched for endogenous peroxidase, and subjected to antigen retrieval by 15 min of microwave boiling. Sections were incubated overnight at 4°C with primary antibodies (anti-Ki67 1:100, anti-TOP2A 1:500, or anti-CD138 1:75), visualized with EnVision-HRP/DAB+ and hematoxylin, and scanned (×40 resolution). Antibody source and catalog details are provided in the key resources table.

#### Multiplex mRNA *in situ* hybridization

FFPE sections (4 μm) were stained using the RNAscope Multiplex Fluorescent Reagent Kit v2 according to the manufacturer’s protocol, with DAPI incubation extended to 3 min and washing in buffer prior to mounting. Sections were hybridized with probes targeting human IGHA1-A2, IGHM, and pan-IGHG, followed by signal amplification using TSA Vivid fluorophores at dilutions of 1:1500 (520-IgA and 570-IgM) and 1:10 000 (650-IgG).

### Quantification and statistical analysis

#### General

Statistical analyses were performed in R^38^ (v4.4), with visualization using the ggplot2,^39^ ggridges,^40^ and patchwork packages.^41^ All *p* values were adjusted (Padj) for multiple comparisons using the methods stated in the relevant section and figure legends. A complete overview of the software tools is provided in the key resources table.

#### Demographic and clinical variables

Continuous variables were tested for normality with the Shapiro–Wilk test and visually assessed with QQ-plots. Welch’s test was used for normally distributed variables and the Mann–Whitney U test for non-parametric and ordinal variables. Categorical variables were analyzed using contingency tables, and proportional differences between groups were tested using Fisher’s exact test.

#### Serum multiplex analysis

Linear mixed-effects model (LMM) accounting for repeated measurements was implemented via lme4.^18^ Cytokine type and response status were modeled as interacting fixed effects and donor as a random effect. Residual normality and homoscedasticity were verified.^44^ (Figures S2A and S2B). The emmeans package^43^ was used to compute marginal means and contrasts with the Kenward–Roger degrees of freedom adjustment. Multiple comparisons were false discovery rate (FDR)-corrected (Benjamini–Hochberg). Multivariate cytokine patterns were explored and visualized using PCA (FactoMineR,^45^ factoextra^46^) and PLS-DA (mixOmics^47^), using a two-component model on responder/non-responder samples only. Variable importance in projection (VIP) scores identified discriminatory cytokines (threshold >1.0). All analyses used natural-log-normalized, Z-score-transformed data.

#### Upstream RNA sequencing processing

FASTQ file quality was assessed with FastQC, then reads were filtered and trimmed with fastp and aligned to the genome reference with STAR for quality control. Quality metrics were extracted with Picard CollectRNASeqMetrics, and transcript counts were generated using Salmon quasi-mapping against the GRCh38 transcriptome reference. One responder inflamed lamina propria sample was excluded for technical reasons owing to a low gene count (10,000 versus the expected 30–50 million reads). Transcript counts were imported into R and aggregated to gene counts using tximport.

#### Differential expression analysis

Gene counts were normalized and analyzed for differential expression using the DESeq2 Bioconductor package with thresholds of Padj <0.05 and |log2 fold change (log2FC)| > 0.3, including only protein-coding genes. Intra-gene sample differences were visualized using normalized counts (median of ratios), whereas multivariate analyses used variance-stabilized transformed data.

#### TNF pathway expression analysis

To compare the expression of *TNF*, *TNFRSF1A*, and *TNFRSF1B* between non-responders and responders, an LMM was fitted for each gene in each tissue compartment using the emmeans package^20^: expression ∼ inflammation_status_× response_status_+ (1|donor). Model residuals were inspected for normality and homoscedasticity. Multiple testing was adjusted using the ‘mvt’ method based on the multivariate t distribution within each analysis. Models with a significant interaction were analyzed with simple pairwise fixed effects, while models without a significant interaction were analyzed for main effects.

#### Enrichment analyses

Over-representation enrichment analysis used thresholds of Padj <0.05 and |log2FC| > 0.3. We used DAVID,^53^^,^^54^ MetaCore, and the gprofiler2 package^55^ with Gene Ontology Biological Processes, Reactome, and KEGG as annotated references, in addition to MetaCore process networks. Multiple comparisons were adjusted using Benjamini–Hochberg or g:SCS (gprofiler2-specific).

#### Weighted gene co-expression network analysis

WGCNA^56^ was used to identify clusters of co-expressed genes (modules) in the epithelial samples (*n* = 22), lamina propria samples (*n* = 21), and uninflamed lamina propria (*n* = 10). Variance-stabilizing transformed (VST) expression values were used as input. Hierarchical clustering did not detect sample outliers requiring exclusion. For each dataset, a signed hybrid network was constructed using Pearson correlation, with the soft-thresholding power selected as the lowest value yielding a scale-free topology fit of R^2^ > 0.80. A topological overlap matrix was then computed, and genes were hierarchically clustered using average linkage. Modules were identified from the resulting dendrogram using the dynamic tree-cut algorithm with a minimum module size of 50 genes, and modules with highly correlated eigengenes were merged at a cut height of 0.15 (corresponding to an eigengene correlation ≥0.85). Each module eigengene was defined as the first principal component of the module’s expression profile. Module–trait relationships were assessed by Pearson correlation between module eigengenes and sample traits, with significance evaluated using Student’s asymptotic *p*-values. Enrichment analysis was performed on trait-correlated modules using gprofiler2^55^ with Gene Ontology, Reactome, and KEGG pathways, considering Padj <0.01 as significant. Modules with biologically meaningful enrichment were retained.

#### Assessment of glucocorticosteroids as a potential confounder

Among donors profiled by laser microdissection and RNA-sequencing, baseline corticosteroid use was compared between response groups using a two-sided Fisher’s exact test. To evaluate whether corticosteroid use could account for the humoral signature identified in uninflamed lamina propria, we performed three complementary analyses on the uninflamed lamina propria samples (Table S8). First, expression of the mediumorchid module genes (*n* = 63) and a curated plasma-cell reference gene set (*n* = 19) was visualized stratified by response status and baseline corticosteroid use. Second, the module eigengene—the first principal component of the module gene expression, computed with the module Eigengenes function in the WGCNA package, was modeled by ordinary least squares as a function of response status, corticosteroid use, and both terms jointly, with coefficients reported as effect estimates and 95% confidence intervals. Third, three glucocorticoid-response gene sets, a curated set of canonical glucocorticoid-induced genes and the MSigDB^62^^,^^63^ Gene Ontology Biological Process sets GOBP_RESPONSE_TO_CORTICOSTERONE and GOBP_RESPONSE_TO_CORTICOSTEROID (MSigDB v2025.1.hs), were scored per sample by single-sample gene set enrichment analysis (ssGSEA) using the GSVA package, applied to the same normalized expression matrix used for network construction. The association between the module eigengene and each glucocorticoid-response score was assessed by Spearman correlation, and the gene-level overlap between the module and each glucocorticoid-response set was quantified. All analyses were performed in R v4.4.

#### Cell type deconvolution

Colonic single-cell RNA sequencing data from Thomas et al.^10^ (815,900 cells; GEO: GSE282122) were used as the reference dataset. Custom references were created for lamina propria and epithelial samples (Table S2). Cell-type-specific profiles were derived using pseudobulk expression normalized to counts per million (CPM) used to build signature matrices in CIBERSORTx,^58^ optimized separately for epithelial and lamina propria samples. Deconvolution was performed using the CIBERSORTx HiRes module with default parameters, except for B-mode batch correction,^57^ on epithelial (*n* = 22) and lamina propria (*n* = 21) samples separately. Cell-type proportions were treated as compositional data summing to unity, and zero values were adjusted by adding a constant (half the smallest non-zero value) to all proportions. Differences between inflamed and uninflamed samples were tested using multivariate analysis of variance on additive log-ratio-transformed data, followed by LMM to account for donor pairing. Multiple testing was corrected using Benjamini–Hochberg, with significance defined at FDR <0.05. Centered log-ratio-transformed data were used for visualization.

#### Pseudobulk validation

Single-cell RNA sequencing data from the independent longitudinal anti-TNF treatment cohort Thomas et al.^10^ were used to validate gene expression patterns associated with the non-responder uninflamed lamina propria. By filtering (based on the definitions provided in that dataset) only cells from pre-treatment colonic UC uninflamed lamina propria samples, pseudobulk profiles were generated per patient with either non-remission (*n* = 6) or remission (*n* = 3) status, aggregated for all lamina propria cell types or for IgA plasma cells only (Figure 7A). Normalized counts for genes within the *Cell cycle core* and *Immune system process* networks (Figures 4D and 5C) were plotted for all samples and compared between remission and non-remission status. Differentially expressed genes (Padj <0.05, |log2FC| > 0.3) in uninflamed IgA plasma cells from non-remission versus remission samples were identified using DESeq2. Concordant regulation was defined as median gene expression consistently higher in non-remission/non-responder than in remission/responder patients across both datasets.

#### Immunohistochemistry and multiplex mRNA *in situ* hybridization quantification

Tissue compartments (epithelium versus lamina propria) were segmented using a DeepLabV3+ deep learning model with a ResNet-18 encoder. Validation-set performance was comparable to published colon epithelium datasets, with high accuracy (intersection-over-union >0.95) for epithelium.^60^^,^^61^ Artifacts, necrotic debris, and mucin pools within the segmented lamina propria were manually excluded in QuPath^59^ prior to quantification in uninflamed (*n* = 4/6) and inflamed (*n* = 6/6) samples of non-responders and responders, respectively (Table S9). Owing to dense clustering of plasma cells in the lamina propria, which hampered individual nuclei separation, CD138 was quantified using an intensity-based pixel classifier (DAB-positive pixels per total tissue pixels). For Ki67 and TOP2A, distinct cell nuclei were quantified using watershed-based cell detection (DAB-positive nuclei per total nuclei). Color deconvolution was set manually on a representative image scan. A separate QuPath pixel classifier was trained for initial tissue detection and further characterized for multiple morphological parameters. For multiplex *in situ* hybridization, tissue regions were identified using a trained pixel classifier, and cells were detected within these regions based on the DAPI channel using watershed-based cell detection. Subcellular detection was applied across IgA, IgM and IgG channels to identify marker-specific signals using intensity thresholds selected to separate signals from the background, enabling quantification relative to total nuclei. Normality was assessed using the Shapiro-Wilk test and visually by Q-Q plots. Statistical analyses were performed in GraphPad prism, using two-tailed Welch’s *t* test for normally distributed data and the Mann-Whitney U test for non-normally distributed data.

## Supplemental information

Document S1. Figures S1–S10

Table S1. Serum multiplex analysis input data

Table S2. Single-cell RNA sequencing reference dataset used for cell type deconvolution analysis (A) Cell type reference populations (cell counts for 14 lamina propria and 15 epithelial cell types). (B) Epithelial deconvolution reference signatures. (C) Lamina propria deconvolution reference signatures

Table S3. Serum multiplex linear mixed-effects model (LMM) detailed output

Table S4. Differential inflammatory expression analyses (A) Differential expression analysis: inflamed vs. uninflamed epithelium. (B) Differential expression analysis: inflamed vs. uninflamed lamina propria

Table S5. Protein-coding differentially expressed genes (DEGs) between uninflamed lamina propria R vs. NR

Table S6. Differentially expressed genes (DEGs) between uninflamed IgA plasma cells in R vs. NR from the independent validation cohort (Taurus data)

Table S7. Differentially expressed gene (DEG) analyses of TNF, TNFRSF1A and TNFRSF1B

Table S8. Analysis of glucocorticosteroid as a confounder for humoral signature

Table S9. QuPath analysis parameters for automated Ki67, TOP2A and CD138 cell detection and quantification
