# Supplementary material for: Uninflamed colonic tissue harbors humoral activity linked to anti-TNF therapy non-response in ulcerative colitis
Source: iScience. 2026 Sep 8;29(9):117408. doi: 10.1016/j.isci.2026.117408 (PMC13573676; doi:10.1016/j.isci.2026.117408)
Supplement: Supplementary file 1 [file mmc1.pdf]

iScience, Volume *29*

Supplemental information

Uninflamed colonic tissue harbors humoral activity linked to anti-TNF therapy non-response in ulcerative colitis

Gunnar Andreas Enerhaug Walaas, Silje Sveaas, Lusie Frostvoll Kuraas, Arun Sridhar, Kari Lillebråten, Hanne Fossen, Henrik Sahlin Pettersen, Arnar Flatberg, Atle van Beelen Granlund, Arne Kristian Sandvik, Ann Elisabet Østvik, Torunn Bruland, and Ingunn Bakke

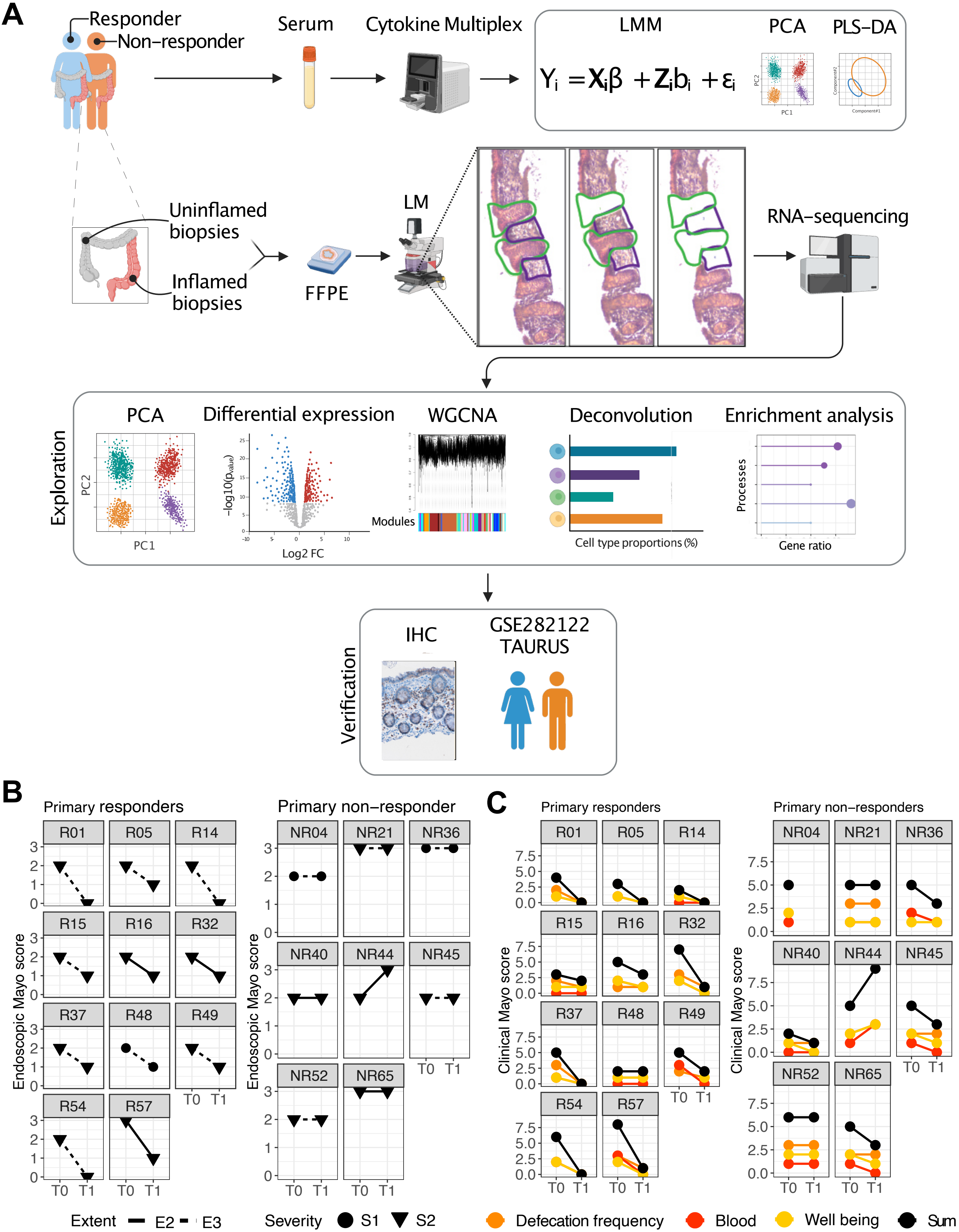

A
Responder
Non−responder
Serum
Cytokine Multiplex
LMM
$Y_i = X_i\beta + Z_i b_i + \varepsilon_i$
PCA
PLS-DA
PC1
PC2
Component#1
Component#2
Uninflamed biopsies
Inflamed biopsies
FFPE
LM
RNA−sequencing
Exploration
PCA
Differential expression
WGCNA
Deconvolution
Enrichment analysis
PC1
PC2
−log10(pvalue)
Log2 FC
Modules
Cell type proportions (%)
Processes
Gene ratio
Verification
IHC
GSE282122
TAURUS
B
Primary responders
Primary non−responder
R01
R05
R14
R15
R16
R32
R37
R48
R49
R54
R57
NR04
NR21
NR36
NR40
NR44
NR45
NR52
NR65
Endoscopic Mayo score
T0
T1
Extent
E2
E3
Severity
S1
S2
C
Primary responders
Primary non−responders
Clinical Mayo score
Defecation frequency
Blood
Well being
Sum

Figure S1. Study design and clinical parameters. Related to Figure 1A and Table1

(A) Study workflow: Serum and colonic biopsies were collected from biologically naïve UC patients with active inflammation before anti-TNF initiation (T0). Serum was analyzed for multiple cytokines. Formalin fixed paraffin embedded (FFPE) biopsies from uninflamed (n=10) and inflamed (n=12) mucosa were laser microdissected (LM) into epithelial (n=22) and lamina propria (n=21) samples for RNA sequencing, processed and analyzed with bioinformatic tools and validated in an independent cohort. Patients classified as responders (R) or non-responders (NR) at 6 months (T1). (B, C) Endoscopic Mayo scores with Montreal classification (B) and Clinical Mayo scores (C) at T0 and T1 for individual patients. E2 = left-sided colitis (line), E3 = extensive colitis (dotted line). S1 = mild UC (circle), S2 = moderate UC (triangle). LMM = linear mixed model. PCA = principal component analysis. PLS-DA = Partial Least Squares Discriminant Analysis. WGCNA = weighted gene co-expression network analysis. IHC = immunohistochemistry. Figure S1A was created in and republished from BioRender under a CC BY license, with permission from Biorender, 2025.

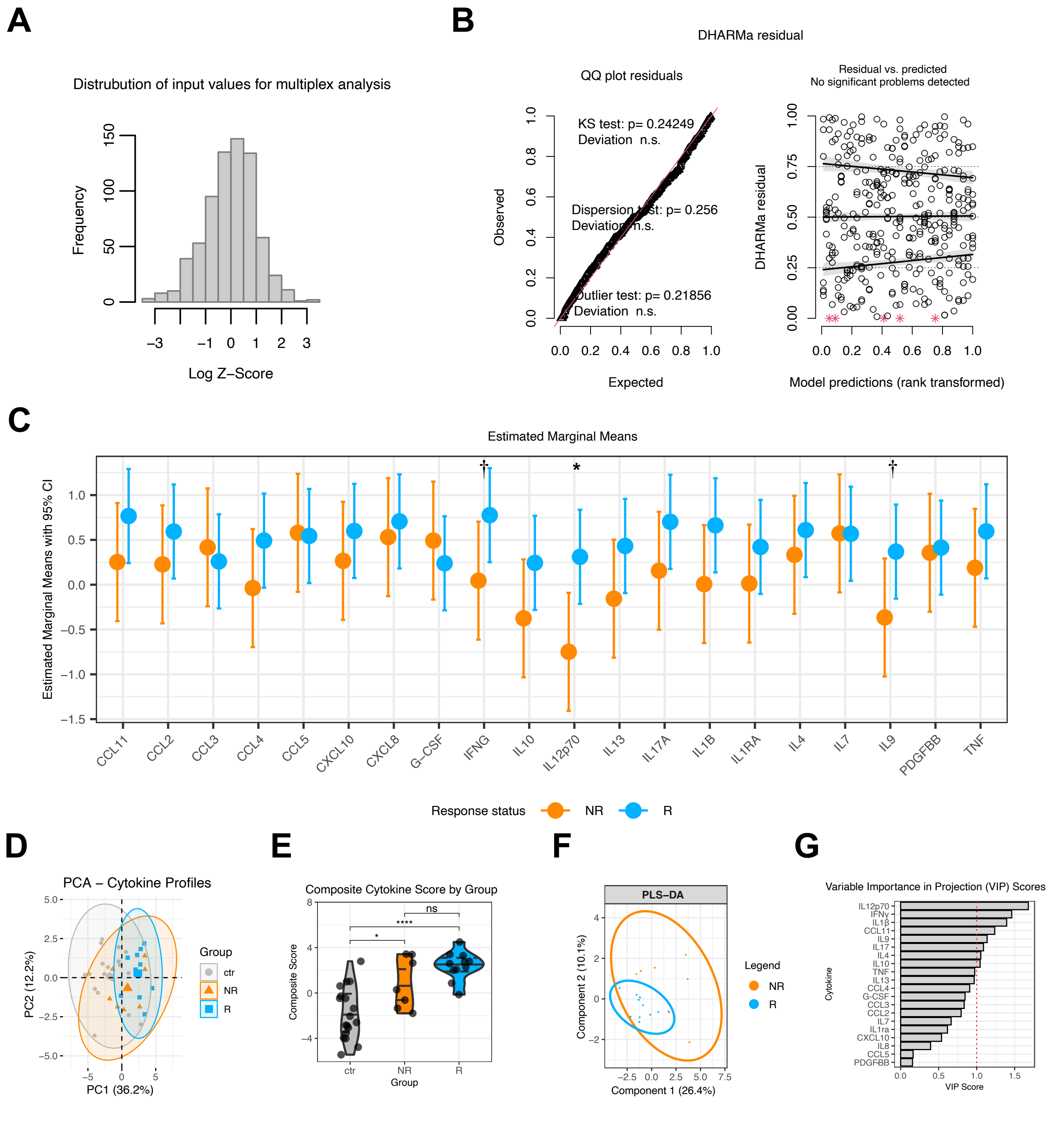
A
Distrubution of input values for multiplex analysis
Frequency
Log Z–Score
B
DHARMa residual
QQ plot residuals
KS test: p= 0.24249
Deviation n.s.
Dispersion test: p= 0.256
Deviation n.s.
Outlier test: p= 0.21856
Deviation n.s.
Observed
Expected
Residual vs. predicted
No significant problems detected
DHARMa residual
Model predictions (rank transformed)
C
Estimated Marginal Means
Estimated Marginal Means with 95% CI
CCL11
CCL2
CCL3
CCL4
CCL5
CXCL10
CXCL8
G–CSF
IFNG
IL10
IL12p70
IL13
IL17A
IL1B
IL1RA
IL4
IL7
IL9
PDGFBB
TNF
Response status
NR
R
D
PCA – Cytokine Profiles
PC2 (12.2%)
PC1 (36.2%)
Group
ctr
NR
R
E
Composite Cytokine Score by Group
Composite Score
Group
ctr
NR
R
ns
F
PLS–DA
Component 2 (10.1%)
Component 1 (26.4%)
Legend
NR
R
G
Variable Importance in Projection (VIP) Scores
IL12p70
IFNγ
IL1β
CCL11
IL9
IL17
IL4
IL10
TNF
IL13
CCL4
G-CSF
CCL3
CCL2
IL7
IL1ra
CXCL10
IL8
CCL5
PDGFBB
Cytokine
VIP Score

Figure S2. Multivariate serum cytokine analysis. Related to Figures 1A and 1B

(A) Distribution of log and Z-score transformed serum cytokine values. (B) Linear mixed model diagnostics (DHARMa package) Q-Q plot showing theoretical vs observed quantiles with Kolmogorov-Smirnov test results; residual plot with standardized residuals vs predicted values with empirical (solid lines) and theoretical quantiles (dashed lines). (C) Individual cytokine estimated marginal means (±95% CI) from linear mixed model. Responders (blue), non-responders (orange). *Padj < 0.05, †Padj < 0.1. (D) PCA of cytokine profiles with 95% confidence ellipses: controls (grey), responders (blue squares), non-responders (orange triangles). (E) Cytokine composite scores (PC1) by group, analyzed with Wilcoxon test. (F) PLS-DA score plot showing responder/non-responder separation with 95% confidence ellipses. (G) Variable importance in projection (VIP) scores from PLS-DA; red line indicates VIP score = 1.0. PCA = principal component analysis. PLS-DA = Partial Least Squares Discriminant Analysis. *Padj < 0.05, ****Padj < 0.0001, ns = non-significant.

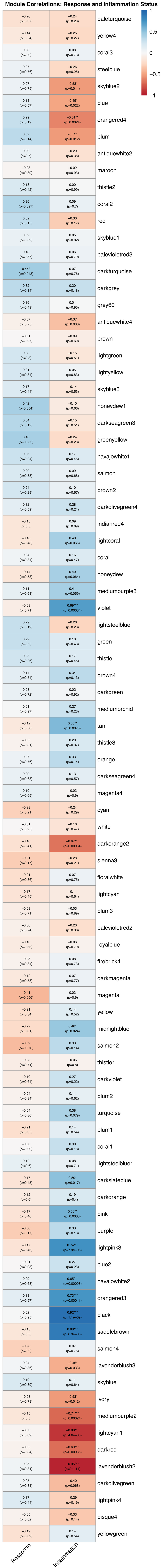

Module Correlations: Response and Inflammation Status
1
0.5
0
−0.5
−1
paleturquoise
yellow4
coral3
steelblue
skyblue2
blue
orangered4
plum
antiquewhite2
maroon
thistle2
coral2
red
skyblue1
palevioletred3
darkturquoise
darkgrey
grey60
antiquewhite4
brown
lightgreen
lightyellow
skyblue3
honeydew1
darkseagreen3
greenyellow
navajowhite1
salmon
brown2
darkolivegreen4
indianred4
lightcoral
coral
honeydew
mediumpurple3
violet
lightsteelblue
green
thistle
brown4
darkgreen
mediumorchid
tan
thistle3
orange
darkseagreen4
magenta4
cyan
white
darkorange2
sienna3
floralwhite
lightcyan
plum3
palevioletred2
royalblue
firebrick4
darkmagenta
magenta
yellow
midnightblue
salmon2
thistle1
darkviolet
plum2
turquoise
plum1
coral1
lightsteelblue1
darkslateblue
darkorange
pink
purple
lightpink3
blue2
navajowhite2
orangered3
black
saddlebrown
salmon4
lavenderblush3
skyblue
ivory
mediumpurple2
lightcyan1
darkred
lavenderblush2
darkolivegreen
lightpink4
bisque4
yellowgreen
Response
Inflammation

Figure S3. Trait-correlation heatmap from the weighted gene co-expression analysis (WGCNA) of epithelium samples. Related to Figure 2F

Heatmap showing Pearson positive (red) or negative (blue) correlations (Padj in brackets) between epithelial gene modules (>50 genes) and tissue inflammation or response to anti-TNF.

Module Correlations: Response and Inflammation Status

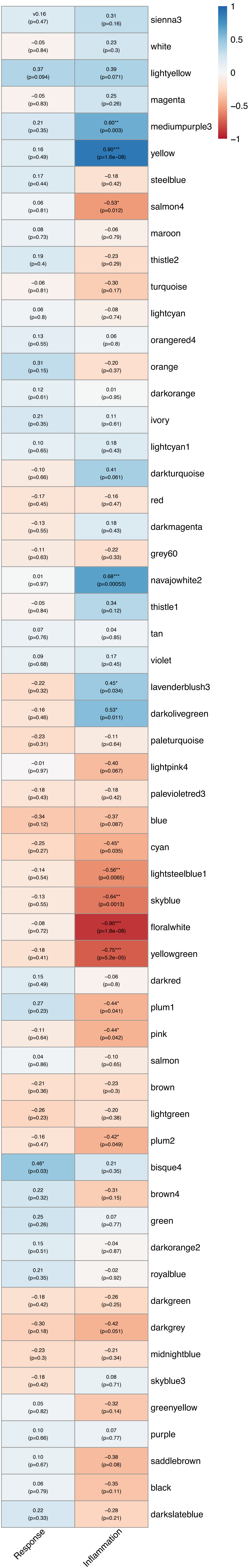

Figure S4. Trait-correlation heatmap from the weighted gene co-expression network analysis (WGCNA) of all lamina propria samples. Related to Figure 3A

Heatmap showing Pearson positive (red) or negative (blue) correlations (Padj in brackets) between lamina propria gene modules (>50 genes) and tissue inflammation or response to anti-TNF.

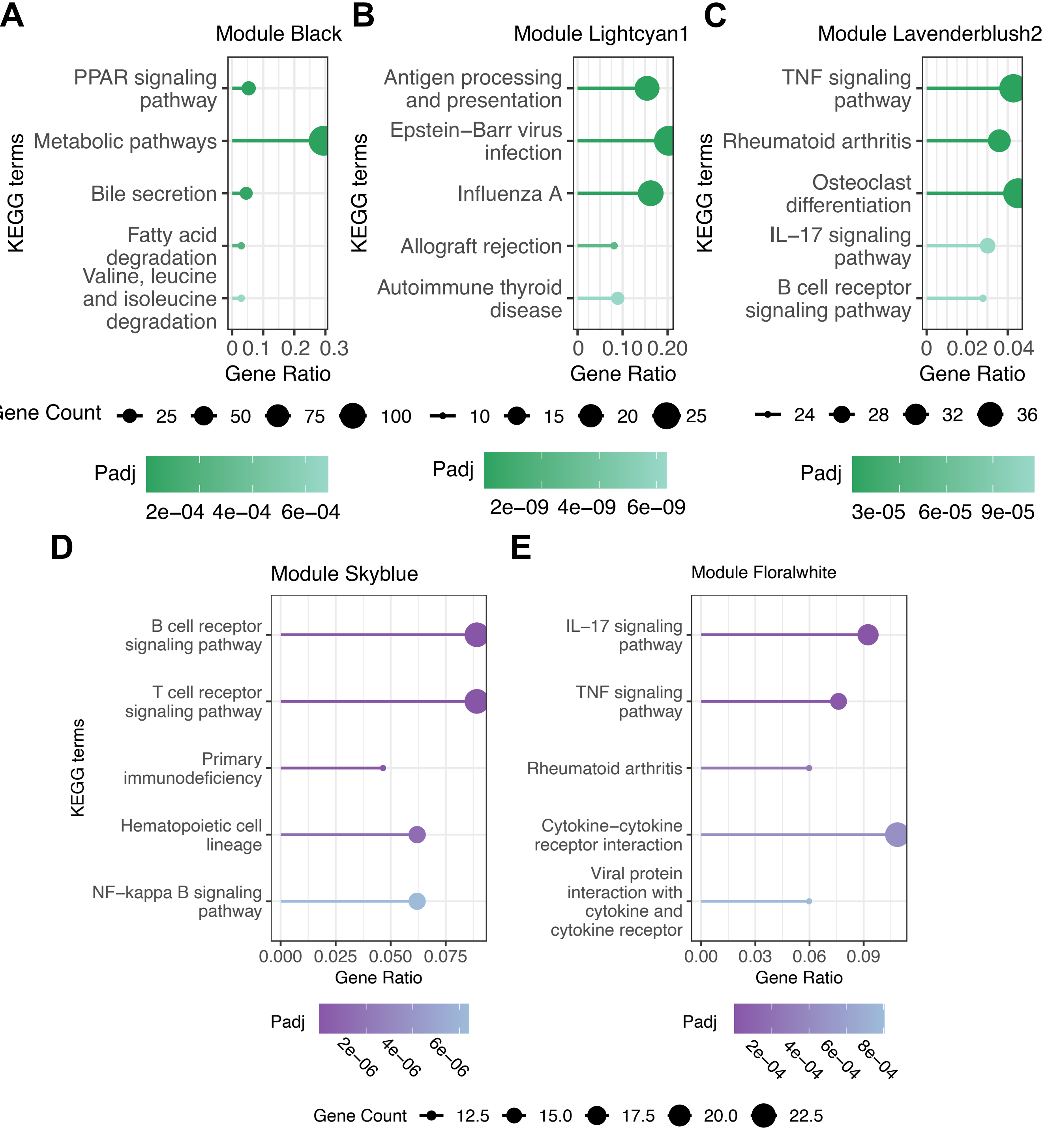

A
Module Black
PPAR signaling pathway
Metabolic pathways
Bile secretion
Fatty acid degradation
Valine, leucine and isoleucine degradation
KEGG terms
0 0.1 0.2 0.3
Gene Ratio
Gene Count 25 50 75 100
Padj 2e−04 4e−04 6e−04
B
Module Lightcyan1
Antigen processing and presentation
Epstein−Barr virus infection
Influenza A
Allograft rejection
Autoimmune thyroid disease
KEGG terms
0 0.10 0.20
Gene Ratio
10 15 20 25
Padj 2e−09 4e−09 6e−09
C
Module Lavenderblush2
TNF signaling pathway
Rheumatoid arthritis
Osteoclast differentiation
IL−17 signaling pathway
B cell receptor signaling pathway
KEGG terms
0 0.02 0.04
Gene Ratio
24 28 32 36
Padj 3e−05 6e−05 9e−05
D
Module Skyblue
B cell receptor signaling pathway
T cell receptor signaling pathway
Primary immunodeficiency
Hematopoietic cell lineage
NF−kappa B signaling pathway
KEGG terms
0.000 0.025 0.050 0.075
Gene Ratio
Padj 2e−06 4e−06 6e−06
E
Module Floralwhite
IL−17 signaling pathway
TNF signaling pathway
Rheumatoid arthritis
Cytokine−cytokine receptor interaction
Viral protein interaction with cytokine and cytokine receptor
0.00 0.03 0.06 0.09
Gene Ratio
Padj 2e−04 4e−04 6e−04 8e−04
Gene Count 12.5 15.0 17.5 20.0 22.5

Figure S5. KEGG enrichment of modules Black, Lightcyan1, Lavenderblush2, Skyblue and Floralwhite. Related to Figures 2F-G, and Figures 3A and 3B

Top enriched KEGG terms (Padj < 0.01) for epithelium specific modules Black (A), Lightcyan1 (B) and Lavenderblush2 (C) and lamina propria specific modules Skyblue (D) and Floralwhite (E). Gene ratio represents the ratio of DEGs *vs* total number of genes in the process networks. The circle size reflects the number of intersecting genes in each network. The color gradient indicates the adjusted p-value (Padj).

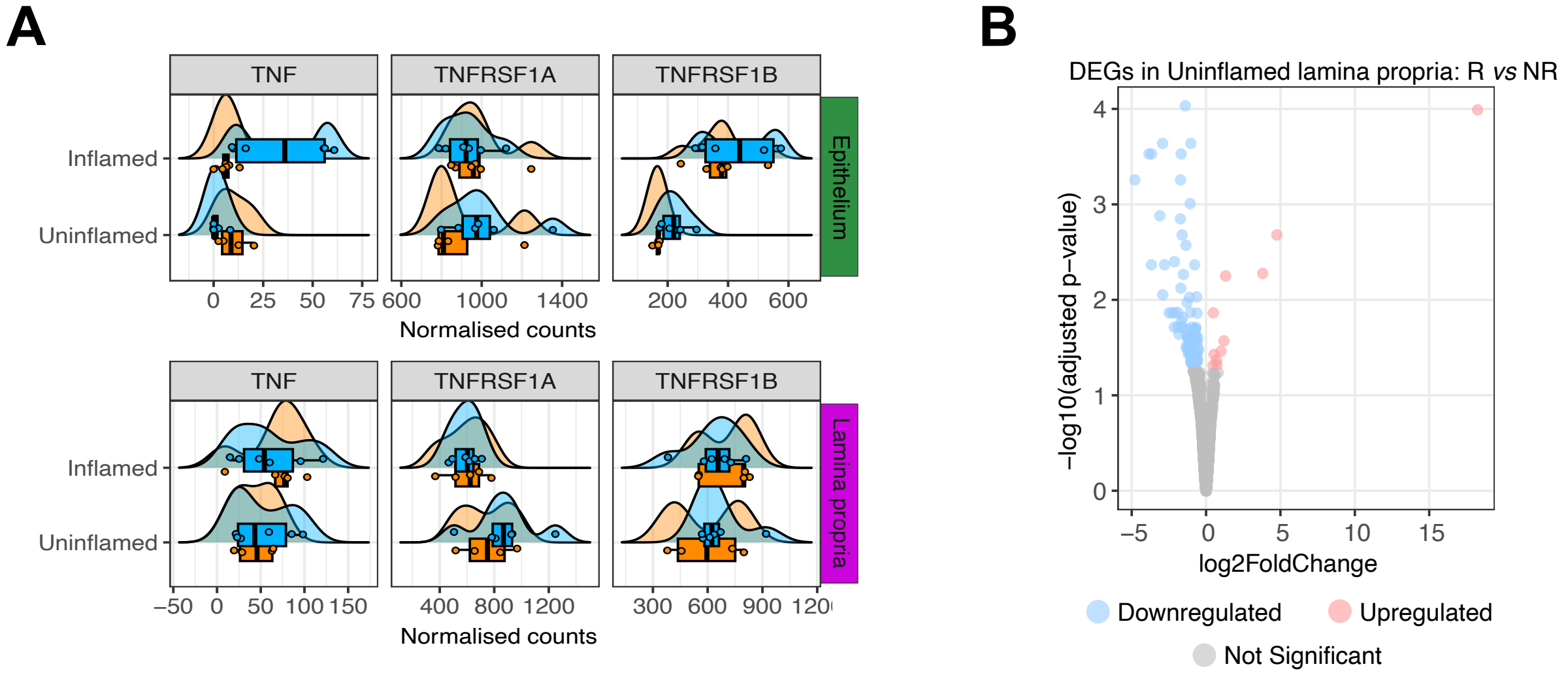
A
TNF
TNFRSF1A
TNFRSF1B
Epithelium
Inflamed
Uninflamed
0 25 50 75
600 1000 1400
200 400 600
Normalised counts
TNF
TNFRSF1A
TNFRSF1B
Lamina propria
Inflamed
Uninflamed
−50 0 50 100 150
400 800 1200
300 600 900 1200
Normalised counts
B
DEGs in Uninflamed lamina propria: R vs NR
−log10(adjusted p−value)
0
1
2
3
4
−5 0 5 10 15
log2FoldChange
Downregulated
Upregulated
Not Significant

Figure S6. **TNF signaling and uninflamed lamina propria DEGs. Related to Figure 4**

(A) Raincloud plots showing inflammation regulation of *TNF*, *TNFRSF1A* (TNFR1), and *TNFRSF1B* (TNFR2) expression in epithelium and lamina propria for responders (blue) and non-responders (orange). Plots include density distribution, boxplot (median±interquartile range), and individual values. (B) Volcano plot of differentially expressed genes (DEGs) between responders and non-responders in uninflamed lamina propria (Padj < 0.05) showing 102 genes upregulated (red) or downregulated (blue).

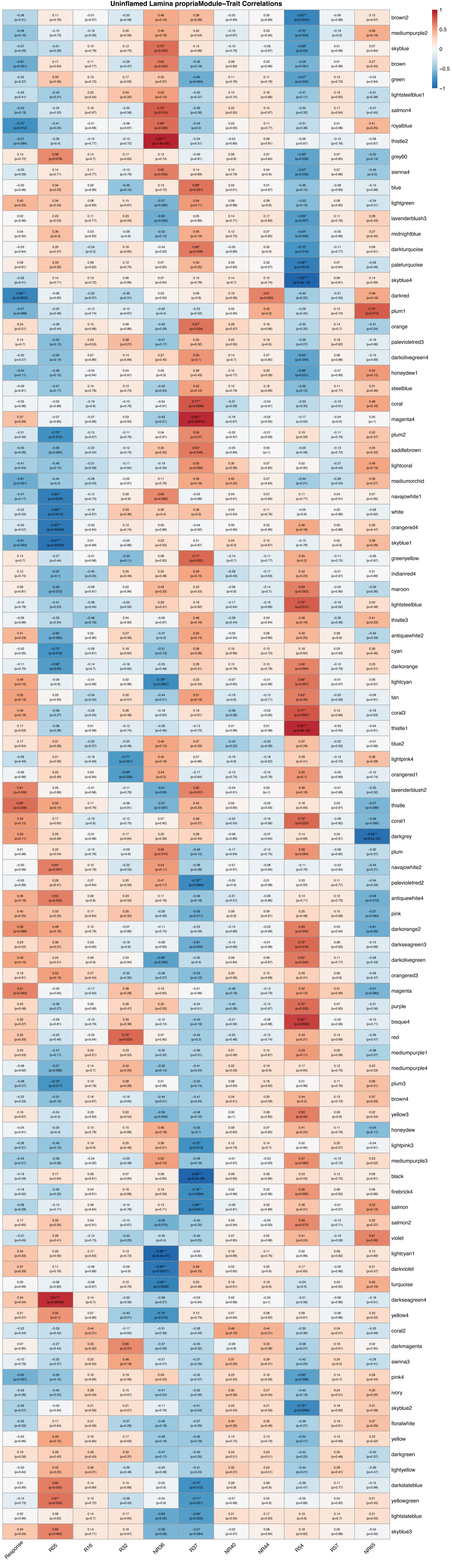
Uninflamed Lamina propriaModule–Trait Correlations
1
0.5
0
−0.5
−1
brown2
mediumpurple2
skyblue
brown
green
lightsteelblue1
salmon4
royalblue
thistle2
grey60
sienna4
blue
lightgreen
lavenderblush3
midnightblue
darkturquoise
paleturquoise
skyblue4
darkred
plum1
orange
palevioletred3
darkolivegreen4
honeydew1
steelblue
coral
magenta4
plum2
saddlebrown
lightcoral
mediumorchid
navajowhite1
white
orangered4
skyblue1
greenyellow
indianred4
maroon
lightsteelblue
thistle3
antiquewhite2
cyan
darkorange
lightcyan
tan
coral3
thistle1
blue2
lightpink4
orangered1
lavenderblush2
thistle
coral1
darkgrey
plum
navajowhite2
palevioletred2
antiquewhite4
pink
darkorange2
darkseagreen3
darkolivegreen
orangered3
magenta
purple
bisque4
red
mediumpurple1
mediumpurple4
plum3
brown4
yellow3
honeydew
lightpink3
mediumpurple3
black
firebrick4
salmon
salmon2
violet
lightcyan1
darkviolet
turquoise
darkseagreen4
yellow4
coral2
darkmagenta
sienna3
pink4
ivory
skyblue2
floralwhite
yellow
darkgreen
lightyellow
darkslateblue
yellowgreen
lightslateblue
skyblue3
Response
R05
R16
R32
NR38
R37
NR40
NR44
R54
R57
NR65

Figure S7. Trait-correlation heatmap from the weighted gene co-expression network analysis (WGCNA) of uninflamed lamina propria samples. Related to Figure 5A

Heatmap showing Pearson positive (red) or negative (blue) correlations (Padj in brackets) between uninflamed lamina propria gene modules (>50 genes) and response to anti-TNF for individual patients.

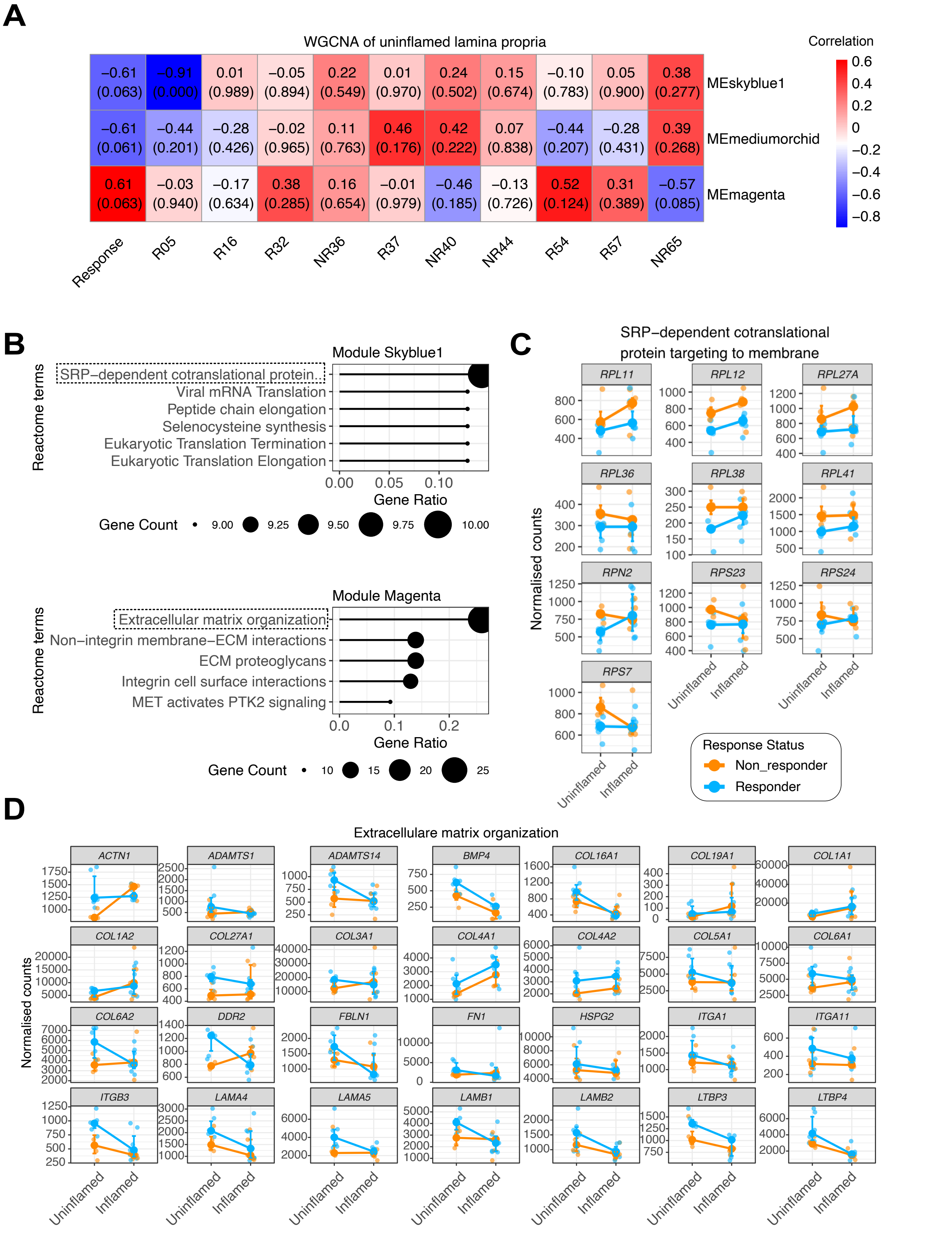

A
WGCNA of uninflamed lamina propria
Correlation
MEskyblue1
MEmediumorchid
MEmagenta
Response
R05
R16
R32
NR36
R37
NR40
NR44
R54
R57
NR65
B
Module Skyblue1
SRP−dependent cotranslational protein...
Viral mRNA Translation
Peptide chain elongation
Selenocysteine synthesis
Eukaryotic Translation Termination
Eukaryotic Translation Elongation
Reactome terms
Gene Ratio
Gene Count
Module Magenta
Extracellular matrix organization
Non−integrin membrane−ECM interactions
ECM proteoglycans
Integrin cell surface interactions
MET activates PTK2 signaling
C
SRP−dependent cotranslational protein targeting to membrane
RPL11
RPL12
RPL27A
RPL36
RPL38
RPL41
RPN2
RPS23
RPS24
RPS7
Normalised counts
Uninflamed
Inflamed
Response Status
Non_responder
Responder
D
Extracellulare matrix organization
ACTN1
ADAMTS1
ADAMTS14
BMP4
COL16A1
COL19A1
COL1A1
COL1A2
COL27A1
COL3A1
COL4A1
COL4A2
COL5A1
COL6A1
COL6A2
DDR2
FBLN1
FN1
HSPG2
ITGA1
ITGA11
ITGB3
LAMA4
LAMA5
LAMB1
LAMB2
LTBP3
LTBP4

Figure S8. Additional analyses of response status-correlated modules from weighted gene co-expression analysis (WGCNA) of uninflamed lamina propria samples. Related to Figure 5A

(A) Heatmap showing Pearson positive (red) or negative (blue) correlations (Padj in brackets) between uninflamed lamina propria gene modules (>50 genes) and response to anti-TNF for individual patients (R# = responders, NR# = non-responders). (B) Reactome enrichment (Padj <0.00001) for modules Skyblue1 (non-responder-associated) and Magenta (responder-associated). Gene ratio represents the ratio of differentially expressed genes vs total number of genes in the process networks. The circle size reflects the number of intersecting genes in each network. (C, D) Expression of genes in (C) *SRP-dependent co-translational protein targeting* and (D) *Extracellular matrix organization* pathways. Median±interquartile range shown with individual samples connected between inflammation states.

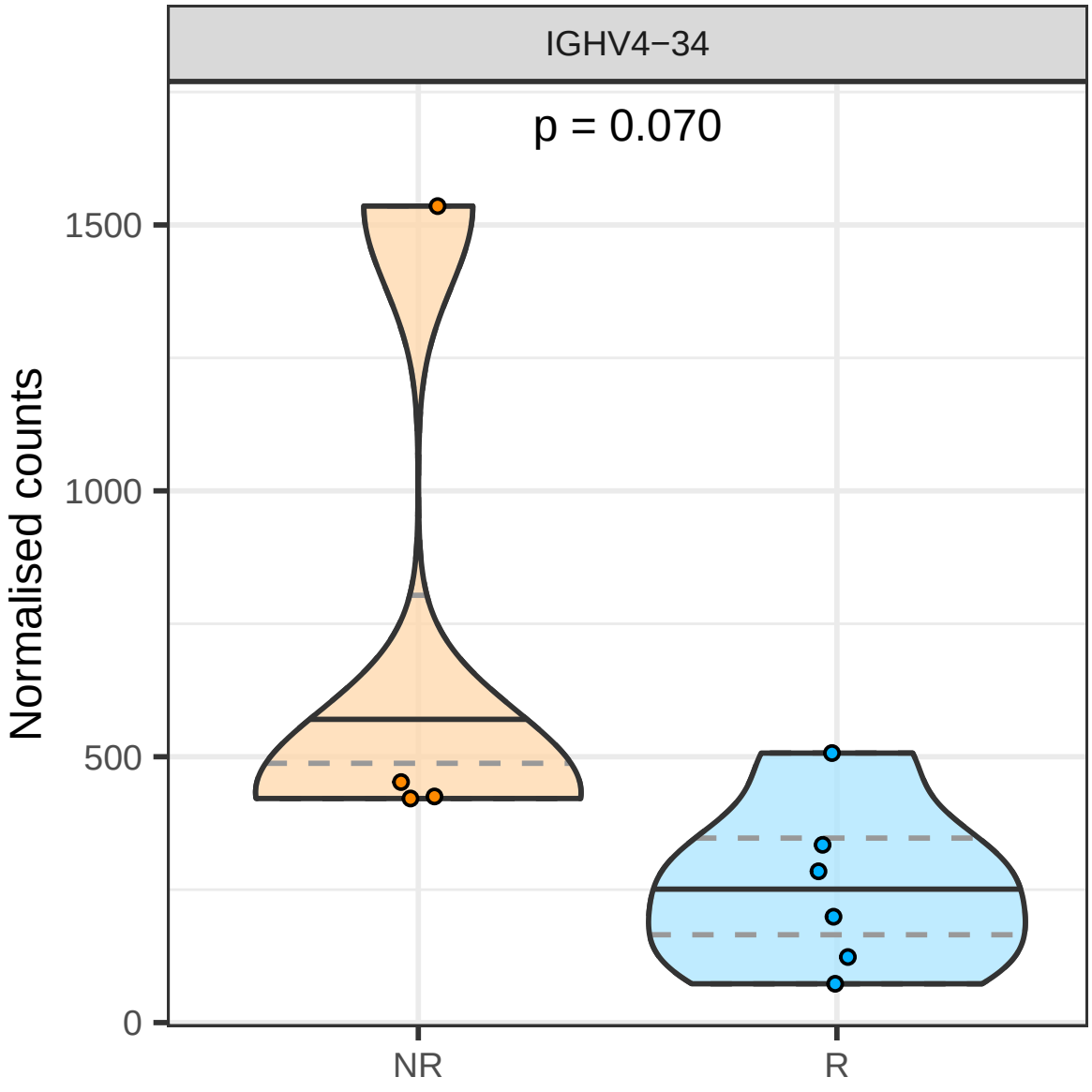

IGHV4−34
p = 0.070
Normalised counts
1500
1000
500
0
NR
R

Figure S9. ***IGHV4-34*** **expression in uninflamed lamina propria. Related to Figure 5**

Violin plot of *IGHV4-34* normalized counts in responders (blue) vs non-responders (orange). Wilcoxon signed-rank test.

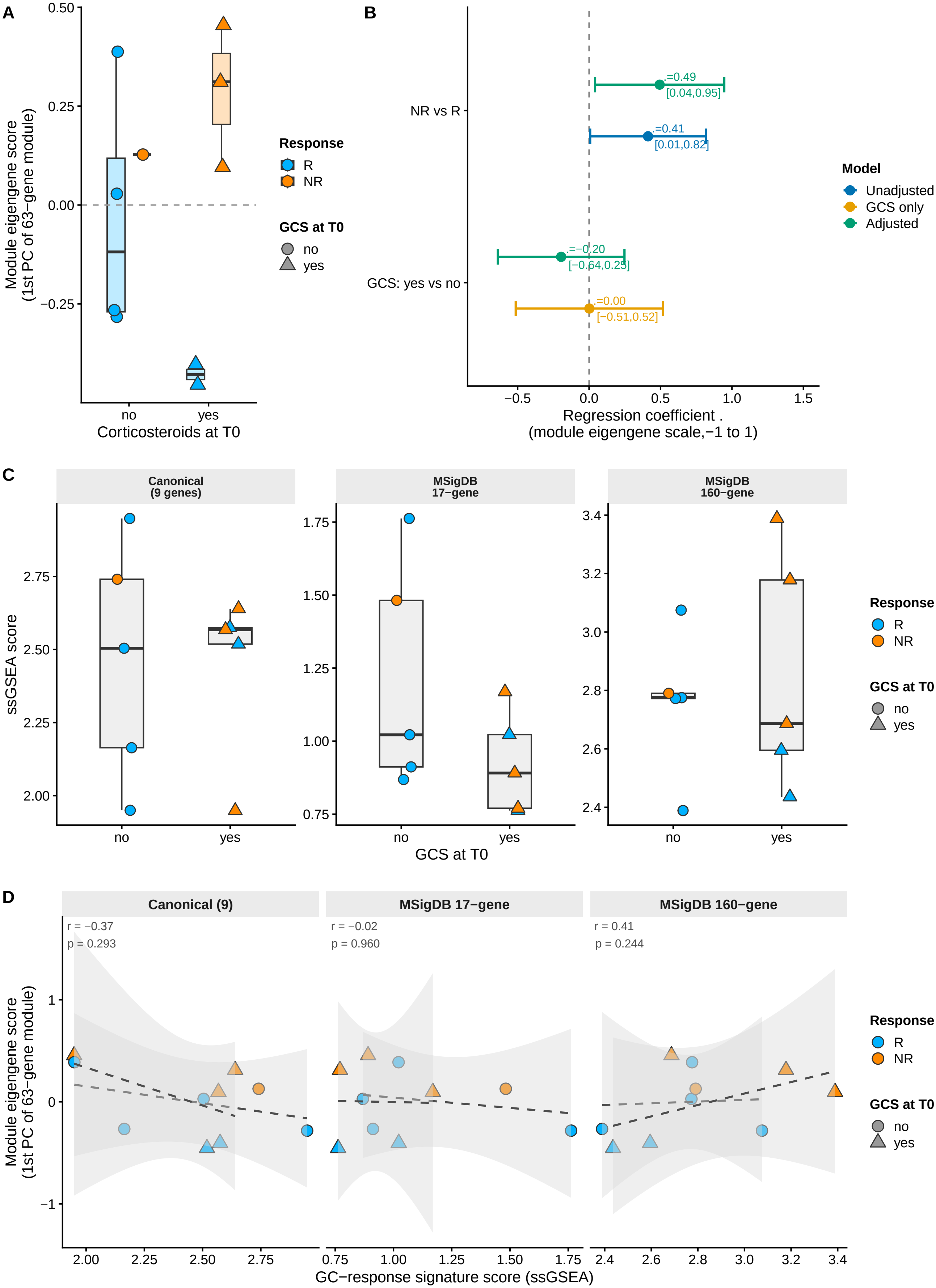

A
B
C
D
Module eigengene score (1st PC of 63−gene module)
Corticosteroids at T0
no
yes
Response
R
NR
GCS at T0
no
yes
NR vs R
GCS: yes vs no
=0.49 [0.04,0.95]
=0.41 [0.01,0.82]
=−0.20 [−0.64,0.25]
=0.00 [−0.51,0.52]
Regression coefficient .
(module eigengene scale,−1 to 1)
Model
Unadjusted
GCS only
Adjusted
Canonical (9 genes)
MSigDB 17−gene
MSigDB 160−gene
ssGSEA score
GCS at T0
Canonical (9)
MSigDB 17−gene
MSigDB 160−gene
r = −0.37
p = 0.293
r = −0.02
p = 0.960
r = 0.41
p = 0.244
GC−response signature score (ssGSEA)

Figure S10. Baseline corticosteroid use does not account for the humoral (module mediumorchid) signature in uninflamed lamina propria of non-responders. Related to Figure 5

(A) Module eigengene (ME) score per donor stratified by baseline glucocorticosteroids (GCS) use. Each point is one donor, coloured by response (R, blue; NR, orange) and shaped by GCS (no, circle; yes, triangle); boxes show median and interquartile range. The GCS-free NR retains an elevated ME, while the two GCS-treated R retain low ME. Thus, the pattern follows response, not steroid exposure. (B) Forest plot of ordinary-least-squares coefficients (β, ME units; 95% CI) for response and GCS, fitted unadjusted, GCS-only, and mutually adjusted. GCS alone explains essentially no ME variance ($\beta \approx 0.00$); the response coefficient is stable and increases after adjustment ($0.41 \rightarrow 0.49$); the adjusted GCS coefficient is negative (−0.20), i.e. directed toward suppression. Wide CIs are expected at $n = 10$ — the analysis assesses direction and stability, not significance. (C) Positive control. Single-sample gene set enrichment analysis (ssGSEA) scores for three glucocorticoid-response gene sets (canonical 9-gene; MSigDB GOBP_RESPONSE_TO_CORTICOSTERONE, 17 genes; GOBP_RESPONSE_TO_CORTICOSTEROID, 160 genes) by GCS status, indicating that the expression data registers a glucocorticoid transcriptional effect in the expected direction. (D) Spearman correlation between the mediumorchid ME and each GCS-signature ssGSEA score (r and p annotated; dashed line, linear fit; shaded band, 95% CI). Correlations are near-zero or negative for the canonical and 17-gene sets and non-significant throughout, indicating the module does not co-vary with glucocorticoid-response activity. Gene-level overlap between the module and the three GC sets was 0/9, 0/17, and 2/128 (BMI1, BMP6).
